# Does learning history shape the associability of outcomes? Further tests of the outcome predictability effect

**DOI:** 10.1371/journal.pone.0243434

**Published:** 2020-12-18

**Authors:** Wei Liu, Evan J. Livesey, Harald Lachnit, Hilary J. Don, Anna Thorwart

**Affiliations:** 1 Department of Psychology, Philipps-Universität Marburg, Marburg, Germany; 2 School of Psychology, University of Sydney, Sydney, Australia; Universidad Autonoma de Madrid, SPAIN

## Abstract

In recent years, several studies of human predictive learning demonstrated better learning about outcomes that have previously been experienced as consistently predictable compared to outcomes previously experienced as less predictable, namely the *outcome predictability effect*. As this effect may have wide-reaching implications for current theories of associative learning, the present study aimed to examine the generality of the effect with a human goal-tracking paradigm, employing three different designs to manipulate the predictability of outcomes in an initial training phase. In contrast to the previous studies, learning in a subsequent phase, when every outcome was equally predictable by novel cues, was not reliably affected by the outcomes’ predictability in the first phase. This lack of an outcome predictability effect provides insights into the parameters of the effect and its underlying mechanisms.

## Introduction

Learning about the relationship between stimuli and events is a fundamental ability of humans and other animals that enables organisms to prepare for future events and adapt to their environment. A classic example of this ability is Pavlovian conditioning [1]. When a stimulus (the conditioned stimulus, CS, or *cue*) is repeatedly paired with a significant event (the unconditioned stimulus, US, or *outcome*), it comes to elicit a response (the conditioned response, CR) that is appropriate for the imminent delivery of the outcome. Many contemporary theories assume that the CR reflects the organism's prediction of the outcome based on the accumulation of knowledge about the sequential structure of its environment during Pavlovian conditioning [2 for a review]. Associative learning models assume that this knowledge takes the form of associations between mental representations of events, and that learning results in changes to the association between the cue and the outcome (ΔV) [3]. According to the Rescorla-Wagner model, learning is determined by the discrepancy between the outcome experienced, and the outcome predicted by the associations between cues and outcomes (i.e., the *prediction error* is minimized during learning).

Moreover, the Rescorla-Wagner theory includes two fixed parameters that alter the rate of learning and reflect the associability of the cue (*α*) and the associability of the outcome (*β*). According to Rescorla and Wagner, these associabilities are both considered a function of physical characteristics of the cue and outcome respectively, and not subject to changes during learning [4]. However, Mackintosh [5] proposed that the associability of a cue (*α*) can be influenced by its prior learning history. There is now considerable evidence that this is the case. For instance, the *learned predictiveness effect* is a phenomenon in which people learn more rapidly about cues that have previously been experienced as good predictors of an outcome, when they enter into new associations with a novel outcome [6–11]. In contrast, there is little research addressing how the associability of the outcome (*β*) is affected by prior learning.

Recently, several studies in human predictive learning have begun to approach this issue, demonstrating that the extent to which an outcome has been consistently predicted by a set of cues, that is its *previous predictability*, influences new learning about that outcome [12–15, see 16 for a review]. A study conducted by Griffiths, Mitchell, Bethmont and Lovibond [13] first demonstrated an influence of the outcomes’ predictability on later learning with new stimuli in a human causal learning task. Participants were required to learn about the causal relationships between cues (foods) and outcomes (allergic reactions) in a hypothetical scenario (i.e. a fictitious patient) and predict the outcome’s occurrence based on the cues present. Participants who had correctly learned contingencies in Phase 1 learned more rapidly about novel relationships involving previously predictable outcomes than relationships involving previously less predictable outcomes in Phase 2. This *outcome predictability effect* has subsequently been observed in a visual cued search task [12] and in a serial letter-prediction task [14] (see General Discussion for details of these studies). All authors argued that the outcome predictability effect occurs in a manner consistent with the learned predictiveness effect for cues. Hence, the authors suggested that similar mechanisms to those proposed by the Mackintosh model [5] might underlie the outcome predictability effect. In particular, an outcome’s associability (*β*) may vary based on its previous predictability.

It is noteworthy that such an effect of outcome predictability differs from those captured in calculations of prediction error, which form part of the Rescorla-Wagner theory and many others like it [3, 17–20]. Prediction error is determined by the cues that are present when an outcome occurs. Thus, its influence is confined to situations where these cues, or at least similar cues that support strong generalization, are present and the association has been re-activated. It reflects how well an outcome is predicted on a *specific trial* by a *specific cue* configuration. In contrast, we discuss a general influence of the outcome’s predictability on changes to the processing of the outcome itself, independent of the presence of other cues, which will therefore transfer to all new learning situations in which the outcome is present. The outcome predictability effect therefore constitutes a challenge to the assumptions of many traditional associative models of learning and offers new understanding about the role of outcome-processing in associative learning.

Considering the potential theoretical significance of the outcome predictability effect, the present experiments aimed to demonstrate and investigate the effect in a new experimental paradigm, a relatively novel human goal-tracking task [21]. Cue associability effects, in particular the learned predictiveness effect, have been demonstrated in a wide variety of learning paradigms, for example human causal learning [8, 10], but also goal-tracking tasks in animals [6, 7, 9, 11]. If both effects rely on a similar mechanism, we would expect that the outcome predictability effect would also be observed in a wide variety of learning paradigms. The present series of six experiments adapted a goal-tracking task for human participants. Goal-tracking tasks are widely used in animal conditioning studies, [e.g., 22–27], in which animals learn to check (by poking their nose into) a magazine in anticipation of the food outcome when a cue signaling food delivery is presented. Goal-tracking is thought to be representative of the associative structure between the cue and an explicit representation of the outcome, which results in behavior specific to the properties of the outcome, such as its spatial location [28]. Further, it has been shown that goal-tracking behavior is particularly sensitive to the updated value of the outcome and its relationship to the cue [26, 27, 29].

**Fig 1:**
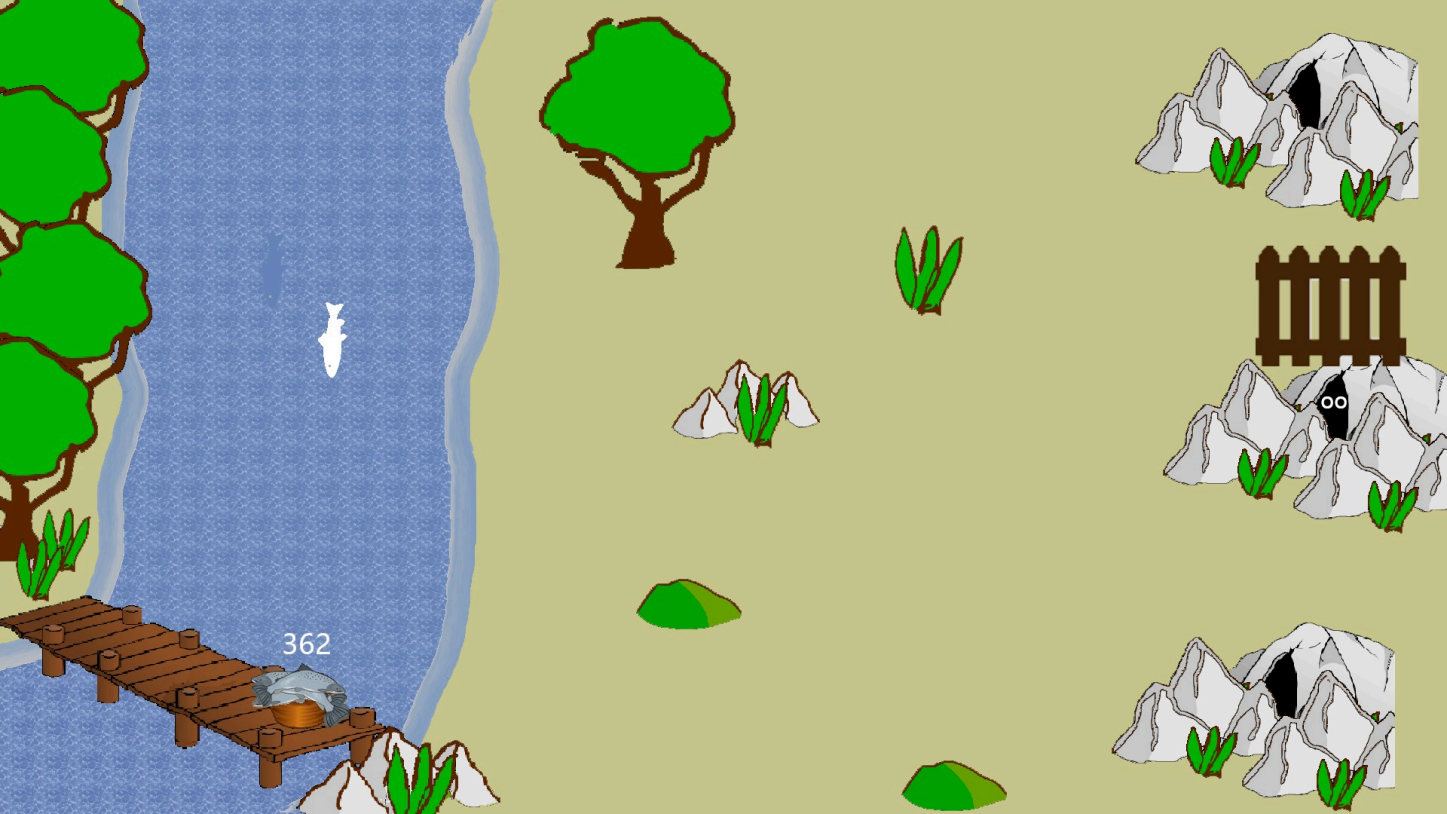
Visual stimuli used in the experiments. Note that not all stimuli shown were present at the same time during the actual experiment.

In the following experiments, we measured human participants’ gaze at a certain goal area in anticipation of a task-relevant outcome. It is well known that eye movements are influenced by predictions and both anticipatory and smooth pursuit eye-movements have been actively investigated in research addressing sequence and motion learning, as well in action and motor control [30, 31]. Koenig and Lachnit [32] reported how the trajectories of saccadic eye movements are affected by memory interference acquired during associative learning. In the present experiments, overt attention was used as an indicator for discrimination learning about cue-outcome relationships where cues preceded different outcomes and predicted their location, identity and timing. The experiments were presented to the participants as a computer game. Participants were instructed that their primary task was to earn points for “fishing” by clicking on the fish that appeared in a river on the left side of the screen (see Fig 1). The secondary task, which was the actual conditioning task, asked participants to feed multiple animals living in several caves on the right side of the screen. The animals could only be fed when their eyes appeared in the mouth of the cave, and participants were required to click on the relevant cave to feed the animal to earn points. The outcome in this case was the appearance of the animal’s eyes in the mouth of the cave, while each cue was a salient audiovisual stimulus that appeared (with varying predictiveness) just before the outcome. For example, the river would turn red before eyes appeared in the top cave, or yellow before eyes appeared in the bottom cave. Outcomes in our study are not inherently significant, but rather acquire control of behavior *during* training by virtue of their task relevance, as participants learned clicking on them would gain game credit. It has been shown in the literature that the two sources of significance have a similar effect on behavior control [33, 34]. Thus, it is reasonable to predict that the outcome with acquired significance are capable of exerting a similar effect as those traditionally used in conditioning research. The appearance of the eyes lasted for only about a second and was difficult to detect using peripheral vision alone, making overt monitoring necessary for performing the task efficiently. Since participants were unable to attend the river and the caves at the same time, learning about the cue-outcome relationship could be determined by their gaze at a specific cave’s entrance in anticipation of an outcome during the corresponding cue.

In every experiment, the outcomes differed in their predictability during initial training in Phase 1. In Phase 2, each outcome became fully predictable by novel cues. In this way, all the properties of the cues and outcomes (cue predictiveness, current outcome predictability and all other aspects of the relationships between the cues and outcomes) were identical. Hence, if learning about the prior predictable and the prior less predictable outcome differed from each other in Phase 2, it should be attributed only to the different predictability of the outcomes learned in Phase 1.

The present study employed three different designs to manipulate outcome predictability during Phase 1 (for an overview see Table 1). In the first three experiments, outcome o1 was consistently preceded by cue A, while the other two outcomes o2 and o3 were each preceded by cue C half of the time, and cue D the other half (Design 1). In this way, o1 was fully predictable, while o2 and o3 were less predictable. Experiment 4 to 6 examined Designs 2 and 3. In Design 2, a partial reinforcement procedure was applied to reduce the predictability of one outcome in initial training. In particular, outcome o2 appeared only half of the time when cue C was presented in Phase 1, and therefore was regarded as partly predictable, while o1 was fully predictable by cue A. In Design 3, outcome o2 was less predictable in the first training phase because it was preceded by cue C half of the time and presented *without* any cue the other half of the time, while o1 was fully predictable by cue A. From prior studies, there is no indication that these different ways of manipulating outcome predictability should affect its influence on subsequent learning. We therefore expected similar outcome predictability effects in all designs.

**Table 1:** Manipulations of outcome predictability in all experiments.

Experiment	Manipulation of outcome predictability in Phase 1
Exp. 1–3	*Design 1*: A→**o1**, A→**o1**, C→o2, C→o3, D→o2, D→o3
Exp. 4 (Group Outcome-absent), Exp. 5	*Design 2*: A→**o1**, C→o2, C→Ø
Exp. 4 (Group Cue-absent), Exp. 6	*Design 3*: A→**o1**, A→**o1**, C→o2, Ø→o2

Note. Letters A, C and D denote cues that were always followed by one outcome, donated as o1, o2 and o3. The absence of the stimuli is denoted as Ø. Outcome o1 is fully predictable with all three designs.

## Experiment 1

Experiment 1 aimed to demonstrate the outcome predictability effect in our human goal-tracking paradigm. In Phase 1, three outcomes differed in their predictability; outcome o1 was consistently predictable and the other outcomes o2 and o3 were only partly predictable (A→o1, A→o1, B→Ø, B→Ø, C→o2, C→o3, D→o2, D→o3). Cue B in Phase 1 as well as cue Z in Phase 2 predicted the absence of any outcome to ensure that participants did not simply shift their attention to the caves at the onset of *any* discrete cue, without learning about the particular relationships between cues and outcomes. In Phase 2, each outcome was fully predictable by a novel cue (W→o1, X→o2, Y→o3, Z→Ø). If the different predictability of outcomes learned in Phase 1 impacts later learning, then learning about o1 should differ from learning about o2 and o3 in Phase 2.

### Methods

#### Participants

Through an a priori power analysis, a sample size of 18 was suggested by G*Power [35] to be sufficient for Design 1 (*F* = 4.54) to detect a medium size effect of outcome predictability (Cohen’s f = .39 [36]) with 80% power (α = .05, two conditions for the within-subjects factor outcome predictability, three groups due to the counterbalancing factor, correlation between repeated measures = .5 as default, no sphericity correction).

Twenty-four undergraduate students from Philipps-Universität Marburg, Germany actually participated in this experiment (*M*_age_ = 23.38 years, age range 19–48 years) in exchange for course credit or payment (EUR € 7). They were allocated equally to the counterbalancing conditions (described below) as they arrived in the experimental room. The studies were approved on the 19 December 2013 by the local ethics committee of the department of psychology, Philipps-Universität Marburg. All participants received a complete description of the experiment and signed a written informed consent form prior to data collection.

Exclusion criteria were (a) missing or invalid gaze data for more than 10% of the total measurements across all training trials and (b) participants who did not gaze at one of the three outcome areas at all during the corresponding cues. Data from two additional participants were excluded based on these criteria.

#### Apparatus and stimuli

All written instructions and visual stimuli were presented on a 23” computer screen and the auditory stimuli were presented with two stereo loud speakers. The experiments were written in Matlab with Psychophysics Toolbox extensions [37]. A Tobii TX300 Eye Tracker measured eye fixations with a frame rate of 300 Hz for both eyes. We used the Tobii Analytics SDK to operate the eye tracker.

Presentation of stimuli on the screen during the learning tasks is illustrated in Fig 1. Color changes of the river from blue to another (red, yellow, green or white) served as visual cues and different sound effects (white noise, pure tone, clicking ringtone, and pulsating "wah-wah" sound) served as auditory cues. Within each learning phase the cues were from the same modality. Assignment of the visual and auditory stimuli to the cues was randomized for each participant. The order of the two modalities was counterbalanced.

Different pairs of symbols (“oo”, “xx” or “++”), representing the eyes of different animals (pig, dog, rabbit) were used as outcomes. The allocation of eye symbols and animal types to outcomes were randomized across participants. The position of the predictable outcome was counterbalanced, resulting in three experimental conditions (see Table 2).

**Table 2:** Cave conditions in Exp. 1.

Cave Condition	Top Cave	Middle Cave	Bottom Cave
1	**o1**	o2	o3
2	o2	o3	**o1**
3	o3	**o1**	o2

*Note*. Each outcome was presented in one of three caves (top cave, middle cave and bottom cave). Position of predictable outcome o1 was counterbalanced across participants.

#### Procedure

Participants were informed that the experiment was going to examine their eye movements during a computer game. After successful calibration and validation of the eye tracking, participants were informed that they had to accomplish two tasks to earn game points: (1) Catching fish by clicking on them with the mouse and (2) feeding the animals when they awake from sleeping by clicking on the eyes that appear on the cave’s entrance. The appearance of the eyes was signaled by visual or auditory cues, such as a change in the color of the river or a certain sound. Participants would gain 2 points for each fish they caught, and would lose 1 point for each fish they missed. Further, participants earned 100 points each time they succeeded in feeding the animal. However, if they missed, they would lose 100 points.

During the entire experiment, two blue fish were always present simultaneously in randomly chosen positions for a maximum of 1.5 seconds. If participants clicked on a fish during its presentation, it would turn white and remain on the screen for 0.83 second. Otherwise, it would disappear and a new blue fish would appear in a new position. The start and end of each learning trial was not explicitly signaled as each trial began with an ITI that varied between 10 and 15 seconds. The cues were then presented for a randomly selected duration between 3.66 to 4.66 seconds, and the outcome (eyes of the animal) appeared during the final second of cue presentation. When participants successfully clicked on the eyes, cartoon images of an animal were shown in animation running from the cave to the river, while the sound of running footsteps was played. If the participant failed to release the animal, the image of a fence appeared above the cave and fell down on the cave, accompanied by the sound of a slamming door. The game score was displayed above the basket of fish and was a constantly updated throughout the experiment.

The experiment consisted of 132 trials. Phase 1 trials were arranged into 12 blocks of eight trials and Phase 2 contained 36 trials grouped into nine blocks. The trial order was randomized within every three blocks and no more than three trials in a row had the same outcome. A drift check controlled the validity of the eye-tracking calibration after each training phase.

#### Data analysis

Only gaze measurements for which both eyes were tracked and identified, were considered as valid. The measurements for the left and right eyes were averaged to obtain the final gaze position. Three goal areas of interest (AOI) were defined as a rectangle around each cave measuring 384 pixels long and 302 pixels wide, centered on the location of the animal’s eyes (the outcome). We then calculated the proportion of valid measurements during a specific time window for which the eye gaze fell within a certain goal area (relative dwell time).

Consistent with magazine training experiments [e.g. 38, 39], we compared this response rate during the presentation of a cue with the response rate immediately before its presentation. The final dependent variable used in the statistical analyses was the proportion of time participants spent looking at the correct goal area during the cue presentation before outcome onset (cue interval) minus the gaze time at the same goal area during an equally long interval before cue onset (pre-cue interval). This measurement is henceforth referred as “dwell time”. Results of additional analyses, in which gaze time during the cue and the pre-cue interval were analyzed separately, can be found in the S1 File.

ANOVAs included the counterbalancing factor “cave condition” as between-subject factor, in order to reduce error variance and increase sensitivity for an effect of outcome predictability. As effects and interactions of this factor are not of interest for the current research question, the corresponding statistical results are reported in S1 File.

Whenever the main analysis revealed non-significant statistics for an outcome predictability effect, a Bayesian method to ANOVA designs was applied to establish the strength of support for the null hypothesis [40]. Bayes factors (BF_01_) were computed based on a Baws method proposed by Mathôt [41], using the software platform JASP for the main effects and interactions of special interest. These “Baws” factors indicate the weight of evidence for *all* candidate models including the effect of interest, compared to the weight of evidence for *all* candidate models without the effect of interest. According to Jeffreys [42], a BF_01_ between one and three provides anecdotal evidence in favor of the candidate model, which in our analyses is always the null hypothesis without the outcome predictability effect. Moreover, a BF_01_ between three and ten provides substantial evidence, between 10 and 30 provides strong evidence, between 30 and 100 very strong evidence, and above 100 decisive evidence in favor of the null hypothesis.

### Results and discussion

#### Phase 1

**Fig 2:**
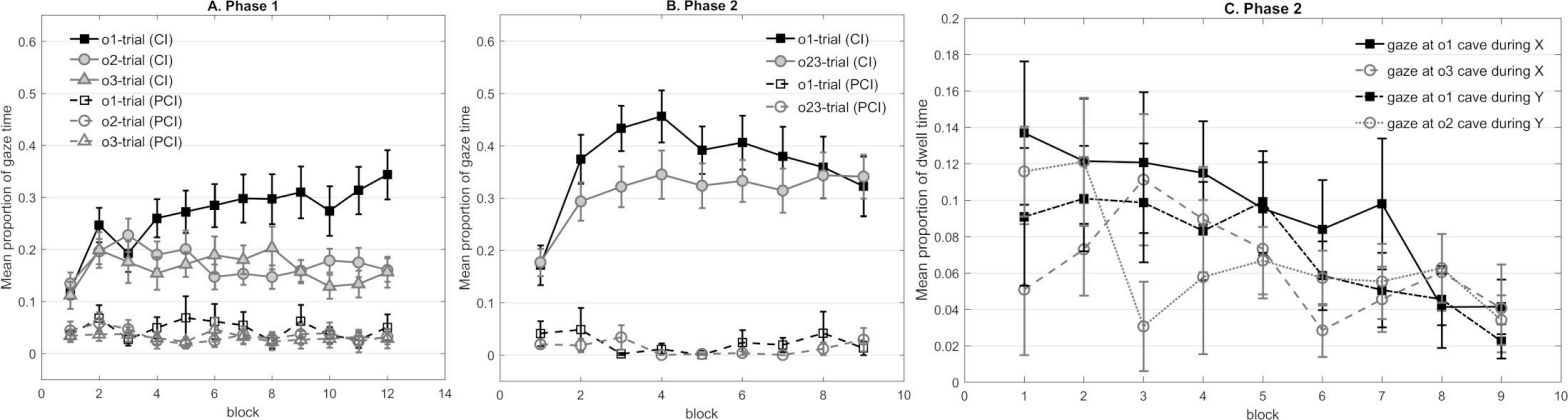
. **Panel A and B represent the mean proportion of gaze time in Experiment 1 that participants looked at the correct outcome’s cave during the cue (CI) and the pre-cue interval (PCI) respectively in each phase** (A) Mean gaze time across the 12 blocks in Phase 1. (B) Mean gaze time across the nine blocks in Phase 2. Note that gaze time in Phase 2 was averaged based on the predictability of each trial’s outcome in Phase 1. Panel C represents the mean *dwell time* (i.e. differences in gaze time during the cue and the pre-cue interval) towards the outcome areas during the cues that had *not* signaled them across nine blocks in Phase 2 (i.e. looking at the o1 cave when Cue X and Y present, looking at o3 cave when X present and at the o2 cave when Y present).

Trials were grouped according to their outcome, resulting in different trial types (e.g. o1-trial, o2-trial or o3-trial), and dwell time in each trial type was averaged within each block. Fig 2A shows that dwell time towards the o1 area in o1 trials increased across blocks, while the correct responses to the cues associated with o2 and o3 remained relatively low. A 3 (outcome: o1 vs. o2 vs. o3) × 3 (cave condition) × 12 (block) mixed design ANOVA was conducted, in which outcome and block were within-subjects factors. The test revealed a main effect of outcome, *F*(2,42) = 7.74, *p* = .001, *η*^*2*^_*p*_ = .269, with significant contrasts regarding the comparison between o1 and both o2 and o3 trials, but not o2 versus o3 (*F*_o1vs.o2_ = 11.03, *p* = .003, *F*_o1vs.o3_ = 8.92, *p* = .007, *F*_o2vs.o3_<1). Notably, a significant outcome × block interaction reflected that dwell time increased more rapidly in o1 trials than in o2 or o3 trials, *F*(22,462) = 2.31, *p* = .022, *η*^*2*^_*p*_ = .099. Further, the main effect of block was significant *F*(11,231) = 3.39, *p* = .007, *η*^*2*^_*p*_ = .139. None of the other main effects and interactions of interest were significant (largest *F* = 1.26, corresponding *p* = .264).

#### Phase 2

Dwell time was averaged within each block based on the outcome’s predictability during Phase 1, resulting in two trial types (trials involving prior predictable outcome and trials involving prior less predictable outcomes). As Fig 2B shows, the anticipatory gaze towards the cave of the previously predictable outcome o1 increased more rapidly across the first four blocks and remained higher than the average dwell time towards the caves of the previously less predictable outcomes. A 2 (outcome predictability: prior predictable vs. prior less predictable) × 3 (cave condition) × 9 (block) mixed design ANOVA was conducted, in which outcome and block were within-subjects factors. The test of dwell time across all nine blocks revealed a non-significant main effect of outcome predictability, *F*(1,21) = 4.12, *p* = .055, *η*^*2*^_*p*_ = .164. Only the main effect of block was significant *F*(8,168) = 6.35, *p* < .001, *η*^*2*^_*p*_ = .232. None of the other main effects and interactions were significant (largest *F* = 1.49, corresponding *p* = .166). On the other hand, the figure shows that maximum anticipation of the outcomes based on their respective cues was already reached within the first half of Phase 2, since the anticipatory responses of both trial types reached the peak in Block 4. As the outcome predictability effect should be strongest while learning was still proceeding, we decided to additionally analyze dwell time across the first half of Phase 2 (Block 1 to 5). This time, the ANOVA demonstrated a significant main effect of outcome predictability, *F*(1,21) = 5.83, *p* = .025, *η*^*2*^_*p*_ = .217. Moreover, the main effect of block was significant *F*(4,84) = 12.83, *p* < .001, *η*^*2*^_*p*_ = .379. None of the other main effects or interactions were significant (largest *F* = 1.17, corresponding *p* = .329).

As expected from the outcome predictability effect, participants exhibited overall longer dwell time in anticipation of the prior predictable than the prior less predictable outcome during the corresponding cue. However, one would also expect this result if participants had developed a general preference for the o1 cave, independently of learning about the novel relationship. To exclude this possibility, we compared the dwell time toward the three caves during cue Z which signaled the absence of any outcome in Phase 2. If a general preference was the reason for the different dwell times in Phase 2, we would anticipate a difference in dwell time between o1 and o2/o3 during Z. A 3 (outcome) × 3 (cave condition) × 9 (block) mixed design ANOVA was conducted. Neither the main effect of outcome, *F*(2,42)<1, nor its interaction with block, *F*<1, was significant, showing no general bias towards any outcome. We observed a significant effect of block, *F*(8,168) = 9.61, *p* < .001, *η*^*2*^_*p*_ = .314. No further main effects or interactions were significant (largest *F* = 1.11, corresponding *p* = .349).

To further discover whether participants had other systematic biases towards a particular outcome, we analyzed the dwell time towards caves o1, o2, and o3 during the cue which did *not* precede them. In particular, we tested whether participants would prefer caves o3 and o2 to cave o1 during cues X and Y, while learning that the latter predicted o2 and o3, respectively (Fig 2C). Such a systematic bias could suggest that participants grouped the outcomes based on the previous predictability, which might impede learning about them as a single outcome in Phase 2. A 2 (cue: X vs. Y) × 2 (previous predictability: o1 vs. o2/o3) × 3 (cave condition) × 9 (block) mixed design ANOVA was conducted. The factor previous predictability yielded a non-significant difference between dwell time towards the area of o1 and o2/o3 during the cue that was not associated with them, *F*(1,21) = 4.08, *p* = .056, *η*^*2*^_*p*_ = .163. The main effect of block was significant, *F*(8,168) = 3.85, *p* = .004, *η*^*2*^_*p*_ = .155. No further main effects or interactions were significant (largest *F* = 2.77, corresponding *p* = .111). In sum, participants did not show any systematic preference for one previously less predictable outcome when learning about the other less predictable outcome in Phase 2.

The present experiment demonstrated that participants successfully learned the cue-outcome relationships in Phase 1, when outcomes differed in their predictability. During the first half of Phase 2, even though all outcomes were completely predictable, participants gazed more at the location of the previously predictable outcome o1 in anticipation of the outcome than the locations of the previously less predictable outcomes (o2 and o3), suggesting that the previously predictable outcome was more readily associated with a novel cue compared to the previously less predictable outcomes (o2 and o3).

We interpret this finding as representing the effect of outcome predictability on later learning. If the effect relies on similar mechanisms to those proposed by Mackintosh [5] and thought to underlie the learned predictiveness effect, our data may indicate that a higher predictability of an outcome can increase its associability and, hence, accelerate learning about its relationship with novel cues in subsequent learning.

It is notable that the effect appeared only in the first half of Phase 2. In particular, a difference in dwell time between two trial types firstly increased and then declined. A possible explanation is that learning about the novel relationships in Phase 2 was completed within the first half of the phase. Afterwards, some participants started to delay switching eye gaze from the distractor task (i.e. fishing) to the goal area (i.e. cave), as they had learned to optimize their motor responses, estimate how long they would need for it and when the outcome would appear at the earliest. The duration of cue-presentation was set between 3.66 to 4.66 seconds based on a pilot study and another learning study [21]. This time span ensures that most participants are able to acquire the cue-outcome associations. However, individual differences still have potential to interfere with the anticipated effect.

If the outcome predictability effect is comparable to a similar learned predictiveness effect anticipated by the Mackintosh model [5], it is reasonable to expect that the effect of outcome predictability would appear during the process of learning, but not necessarily after completing acquisition. If associability *β* of the prior predictable outcome is greater than *β* of the prior less predictable outcome due to Phase 1 learning, this would lead to a rapid increment of its associations. In this fashion, the response to the cue associated with the prior predictable outcome will be greater than to the cue associated with the prior less predictable outcomes during learning. After learning, the associations with all outcomes should be approaching asymptote, so that the difference in response is no longer necessarily evident. Nevertheless, we note the lack of observation of an interaction between outcome predictability and block during Phase 2 learning. One possibility is that the lack of the interaction may be due to early reach of asymptote or a ceiling effect.

An alternative explanation of the observed effect of outcome predictability in the present experiment can be attributed to a blocking effect caused by associations between the context and the outcomes rather than a change in processing of the outcomes themselves. In particular, the presented layout contained many elements forming a context that could also be associated with each outcome. The context therefore has potential to compete for learning in Phase 2. Because o1 was consistently predicted by a cue in Phase 1, the cue is a much stronger predictor of the outcome than is the context, and the contextual association with o1 should therefore be relatively weak. In contrast, o2 and o3 were less predictable in Phase 1, and thus the cues that preceded those outcomes are only marginally more predictive than the context and are far less informative than the cue paired with o1. Therefore, a stronger association between the context and each of those two less predictable outcomes may be established in Phase 1 and then transfer to Phase 2 and preferentially block learning about the novel relationships with o2 or o3. This possibility suggests that the outcome predictability effect might be highly context-specific and motivated the design of Experiment 2, which included a context switch for the experimental groups. That is, if context associations drive the outcome predictability effect, then switching the context between phases should inhibit transfer of context associations, and reduce the effect.

The cave condition interacted significantly with other factors (see S1 File), suggesting a general preference for the outcome in the middle cave. Such a preference cannot explain our key finding, since the predictable outcome o1 occupied the middle cave for only a third of participants and thus if anything, this preference would have worked against observing the effect. Nevertheless, the general preference for the middle cave suggests that the paradigm could be improved in future experiments.

## Experiment 2

**Fig 3:**
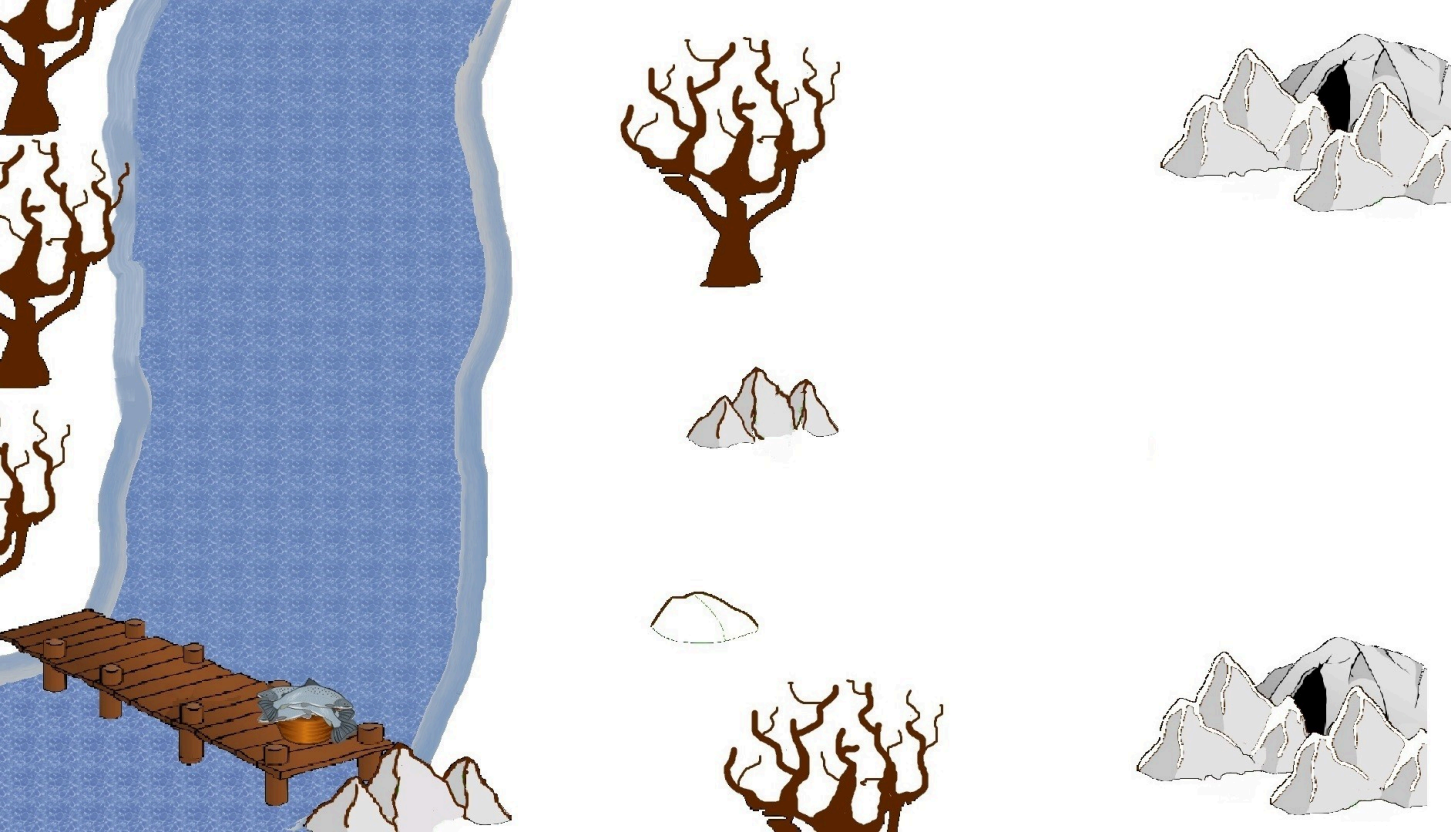
Context “winter” with two caves. Note that the context “Winter” for Shift-3cave had three caves. Contexts differed between the two phases (“summer” vs. “winter”) in Shift-2cave and Shift-3cave.

The present experiment aimed to replicate the findings observed in Experiment 1 and, further, investigate whether the effect is context-dependent. Design 1 was applied again to manipulate outcome predictability. The two experimental groups (*Shift-3cave* and *Shift-2cave*) included a context shift between the two training phases (Layout “summer” versus “winter”, see Fig 3 for the layout winter with two caves), whereas the two control groups (*NoShift-3cave* and *NoShift-2cave*) maintained the same context (i.e. summer layout) present in two phases. In order to minimize the interference of the middle cave advantage, one less predictable outcome (o3) was consistently presented in the middle cave in Phase 1 and this outcome as well as the middle cave were removed in Phase 2 in two of the groups (*Shift-2cave* and *NoShift-2cave)*.

### Methods

Only changes to Experiment 1 are described.

#### Participants

Ninety-six undergraduate students from the Philipps-Universität Marburg, Germany (70 females, 26 males; M_age_ = 22.28 years, age range 18–31 years; 24 in each group) participated in the experiment. Data from ten additional participants were excluded from analysis based on the exclusion criteria.

#### Design, apparatus and stimuli

The stimuli used in Experiment 2 were very similar to those in Experiment 1 with a few exceptions: First, in the *Shift* conditions, a “winter” layout (Fig 3) was displayed during Phase 1, and a “summer” layout was presented in Phase 2, creating a context shift. Second, for the *2cave* conditions, outcome o3 was consistently shown in the middle cave in Phase 1 and both o3 and the cave where it appeared were omitted in Phase 2, leaving two caves and their respective outcomes. Crossing the shift and cave conditions, there were four groups (*Shift-3cave*, *Shift-2cave*, *NoShift-3cave*, and *NoShift-2cave*). Third, auditory stimuli were presented (via ear phones) in Phase 1 for all participants to reduce the number of the counterbalancing conditions. Because we did not observe any statistically significant influence of the cue’s modality in Experiment 1, this manipulation should not impact the demonstration of the outcome predictability effect. Some researchers have suggested that responses to auditory stimuli are faster than to visual stimuli [43, 44]. But even if that were the case, participants should show better learning of all Phase 1 pairings.

#### Procedure

In Phase 2, the 3cave conditions received the same pairings as those in Experiment 1 (W→o1, X→o2, Y→o3, Z→Ø). Each participant in these conditions completed 96 trials grouped into 12 blocks in Phase 1 and 48 trials grouped into 12 blocks in Phase 2. For the 2cave conditions, additional cues were paired with outcomes to slow down the learning process (X→o1, R→o1, Y→o2, S→o2, Z→Ø, T→Ø). In these conditions, there were 96 trials grouped into 12 blocks in Phase 1 and 72 trials grouped into 12 blocks in Phase 2. Moreover, we were concerned that the trial type present on Trial 1 in Phase 2 would affect subsequent learning, if participants learned the cue-outcome relationship in Phase 2 very quickly. Thus, the trial type of the first trial of Phase 2 was counterbalanced across participants.

### Results and discussion

#### Phase 1

**Fig 4:**
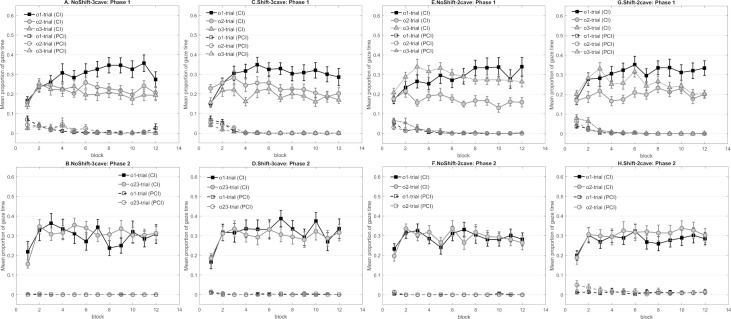
**Panel A to H represent the mean proportion of gaze time in Experiment 2 that participants looked at the correct outcome’s cave during the cue (CI) and the pre-cue interval (PCI) respectively in each phase.** (A) Mean gaze time of NoShift-3cave across the 12 blocks in Phase 1. (B) Mean gaze time of NoShift-3cave across the 12 blocks in Phase 2. (C) Mean gaze time of Shift-3cave across the 12 blocks in Phase 1. (D) Mean gaze time of Shift-3cave across the 12 blocks in Phase 2. (E) Mean gaze time of NoShift-2cave across the 12 blocks in Phase 1. (F) Mean gaze time of NoShift-2cave across the 12 blocks in Phase 2. (G) Mean gaze time of Shift-2cave across the 12 blocks in Phase 1. (H) Mean gaze time of Shift-2cave across the 12 blocks in Phase 2. Note that i) gaze time in Phase 2 was averaged based on the predictability of each trial’s outcome in Phase 1, and ii) o3 as well as the middle cave were not presented in Phase 2 in NoShift-2cave and Shift-2cave.

In Phase 1, all four groups showed longer anticipatory gaze time towards the location of the predictable outcome than the less predictable outcome (Fig 4, top row). Notably, higher responses during o3 trials shown by NoShift-2cave and Shift-2cave confirmed the bias towards the middle cave, since o3 was always presented in the middle cave. Hence, the o3-trial was not included in the analyses for these two groups.

Each group was analyzed individually to determine whether the outcome’s predictability was successfully learned. A 3 (outcome) × 3 (cave condition) × 12 (block) mixed design ANOVA was conducted for the 3cave conditions, while a 2 (outcome) × 2 (cave condition) × 12 (blocks) mixed design ANOVA was conducted for the 2cave conditions. All four groups gazed significantly longer towards the area of o1 than o2 (and o3) during the corresponding cues (NoShift-3cave: *F*(2,42) = 9.08, *p* = .001, *η*^*2*^_*p*_ = .302, *F*_o1 vs. o2_ = 9.10, *p* = .007, *F*_o1 vs. o3_ = 12.43, *p* = .002, *F*_o2 vs. o3_<1; NoShift-2cave: *F*(1,22) = 28.07, *p* < .001, *η*^*2*^_*p*_ = .561; Shift-3cave: *F*(2,42) = 12.47, *p* < .001, *η*^*2*^_*p*_ = .373, *F*_o1 vs. o2_ = 7.66, *p* = .012, *F*_o1 vs. o3_ = 30.13, *p* < .001, *F*_o2 vs. o3_ = 3.59, *p* = .072; Shift-2cave: *F*(1,22) = 32.58, *p* < .001, *η*^*2*^_*p*_ = .597). The test also revealed a significant main effect of block, (NoShift-3cave: *F*(11,231) = 5.58, *p* < .001, *η*^*2*^_*p*_ = .210; NoShift-2cave: *F*(11,242) = 2.84, *p* = .027, *η*^*2*^_*p*_ = .114; Shift-3cave: *F*(11,231) = 6.98, *p* < .001, *η*^*2*^_*p*_ = .249; Shift-2cave: *F*(11,242) = 5.78, *p* < .001, *η*^*2*^_*p*_ = .208). Moreover, the two-way interaction between outcome and block was significant for NoShift-3cave, NoShift-2cave and Shift-3cave, confirming that dwell time towards the area of o1 increased more rapidly than dwell time towards o2 (and o3) across blocks in these groups (NoShift-3cave: *F*(22,462) = 2.40, *p* = .014, *η*^*2*^_*p*_ = .103; NoShift-2cave: *F*(11,242) = 4.80, *p* < .001, *η*^*2*^_*p*_ = .179; Shift-3cave: *F*(22,462) = 2.43, *p* = .017, *η*^*2*^_*p*_ = .104). No further main effects or interactions of interest in each group reached significance (the largest *F*- and the corresponding *p*-value across all groups: *F* = 4.21, *p* = .052).

#### Phase 2

According to the Fig 4B, 4D, 4F, and 4H, none of the four groups showed a difference in dwell time between the trials involving the prior predictable outcome and the trials with the prior less predictable outcomes in Phase 2.

As NoShift-3cave and Shift-3cave, as well as NoShift-2cave and Shift-2cave respectively, contained the same counterbalancing condition and differed only in the application of the context switch, two pairs of groups were compared (NoShift-3cave vs. Shift-3cave, NoShift-2cave vs. Shift-2cave). A 2 (outcome predictability) × 2 (Group Shift vs. NoShift) × 3 (cave condition) × 12 (block) mixed design ANOVA was conducted for the 3cave conditions, while a 2 (outcome predictability) × 2 (Group Shift vs. NoShift) × 2 (cave condition) × 12 (block) mixed design ANOVA for the 2cave conditions. Neither the analysis of NoShift-3cave and Shift-3cave nor the analysis of NoShift-2cave and Shift-2cave demonstrated a difference in dwell time based on the prior predictability of outcomes in Phase 1. Further, neither of the two analyses showed a difference between groups based on the prior predictability of outcomes but only the main effects of block were significant (NoShift-3cave &. Shift-3cave: *F*(11,451) = 7.02, *p* < .001, *η*^*2*^_*p*_ = .146; NoShift-2cave &. Shift-2cave: *F*(11,484) = 6.73, *p* < .001, *η*^*2*^_*p*_ = .133). None of other main effects and interactions of interest in two analyses reached significance (the largest *F*- and the corresponding *p*-value across two analyses: *F* = 2.02, *p* = .057).

Further, the Bayes factors were calculated to assess the non-significant main effect and interactions. For the 3cave conditions, our results provided strong evidence in favor of the models without the main effect of outcome predictability (BF_01_ = 13.33), substantial evidence for the models without the outcome predictability × group interaction (BF_01_ = 3.13), and decisive evidence for the models without the outcome predictability × block interaction (BF_01_ = 250). Very strong evidence was found in favor of the models without the three-way interaction (BF_01_ = 33.33). For the 2cave conditions, the results substantially supported the models without the main effect of outcome predictability (BF_01_ = 8.93) and the models without the outcome predictability × group interaction (BF_01_ = 4.17). We also observed decisive evidence for the models without the outcome predictability × block interaction (BF_01_ = 333.33), and very strong evidence for the models without the three-way interactions (BF_01_ = 52.63).

Although participants in all groups successfully learned the different predictability of outcomes in Phase 1, none of the four groups demonstrated a difference in Phase 2 learning based on the prior predictability of outcomes. It is noteworthy that even the control groups did not replicate the key finding from Experiment 1. Such a result raises the question whether the effect observed in Experiment 1 is reliable. Regarding this matter, it is pointless to discuss the impact of context or the outcome removal if the reliability of the effect remains open. Hence, the next experiment removed the additional manipulations applied in the present experiment, which might be influential on preferential gaze behavior, and aimed to determine whether the effect observed in Experiment 1 is replicable.

## Experiment 3

Since Experiment 2 did not demonstrate the effect of outcome predictability on subsequent learning, we considered the possibility that the effect might not be reliable. Possibly, some additional manipulations applied in Experiment 2, such as the counterbalancing of the outcome presentation in Phase 2, or the change in the number of outcomes might be responsible for the failed observation. Thus, the present experiment is a close replication of Experiment 1.

### Methods

#### Participants

Twenty-four undergraduate students from the Philipps-Universität Marburg, Germany (17 females, 7 males; M_age_ = 22.88 years, age range 19–28) participated in this experiment. Data from four additional participants were excluded from analysis.

#### Stimuli and design

The stimuli and design used in Experiment 3 were the same as in Experiment 1 with only two exceptions: First, the visual cues were shown for Phase 2 across all participants. Second, a drift check was performed after Phase 2 training.

### Results and discussion

#### Phase 1

**Fig 5:**
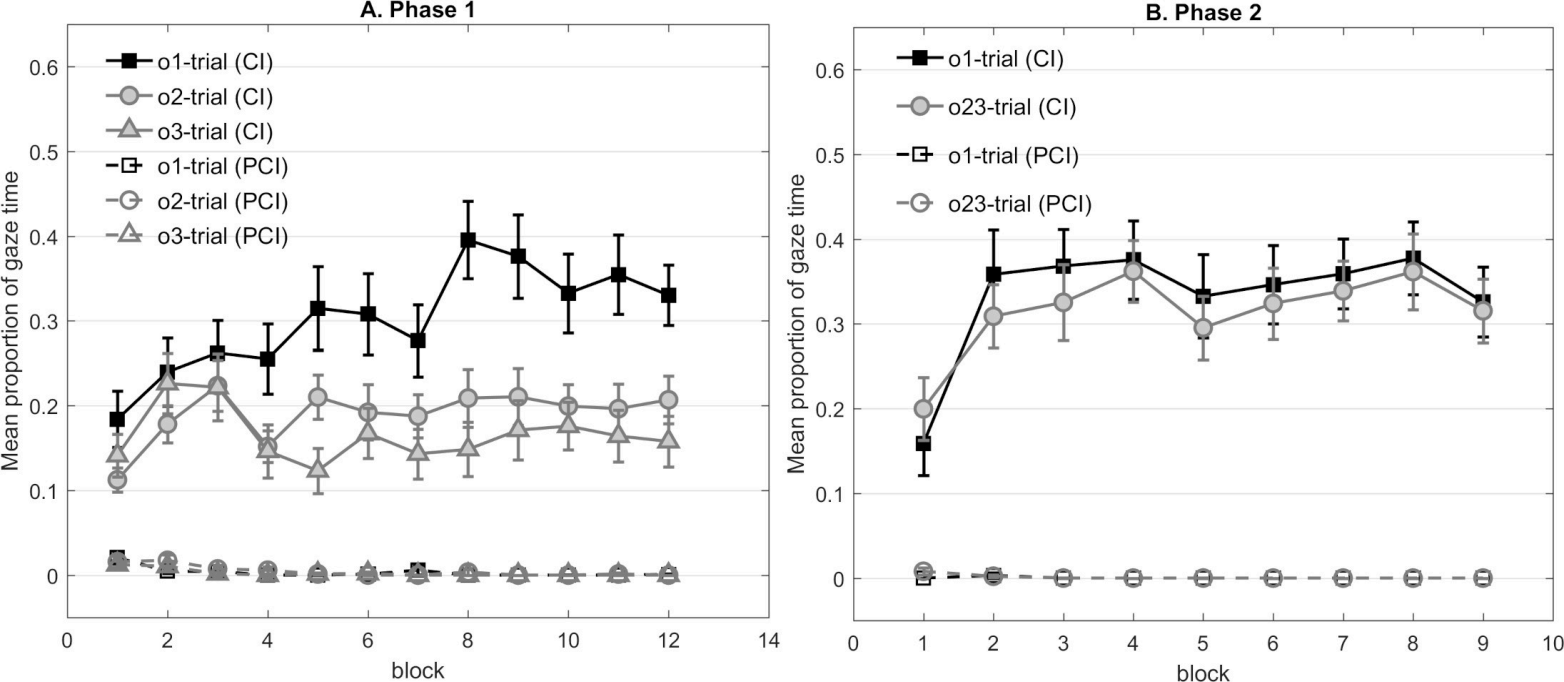
**Panel A and B represent the mean proportion of gaze time in Experiment 3 that participants looked at the correct outcome’s cave during the cue (CI) and the pre-cue interval (PCI) respectively in each phase.** (A) Mean gaze time across the 12 blocks in Phase 1. (B) Mean gaze time across the nine blocks in Phase 2. Note that gaze time in Phase 2 was averaged based on the predictability of each trial’s outcome in Phase 1.

A 3 (outcome) × 3 (cave condition) × 12 (block) mixed design ANOVA was conducted. In line with the illustration in Fig 5A, dwell time towards the area of the predictable outcome o1 was longer than the area of the less predictable outcomes o2 and o3 (main effect of outcome: *F*(2,42) = 21.22, *p* < .001, *η*^*2*^_*p*_ = .503, *F*_o1vs.o2_ = 19.68, *p* < .001, *F*_o1vs.o3_ = 28.41, *p* < .001, *F*_o2vs.o3_ = 2.55, *p* = .125), and this difference increased more rapidly across blocks (outcome × block interaction: *F*(22,462) = 2.81, *p* = .007, *η*^*2*^_*p*_ = .118). Further, the test also revealed a significant main effect of block *F*(11,231) = 4.49, *p* = .004, *η*^*2*^_*p*_ = .176. None of the other main effects and interactions of interest were significant (largest *F* = 1.42, corresponding *p* = .143).

#### Phase 2

A 2 (outcome predictability) × 3 (cave condition) × 9 (block) mixed design ANOVA was conducted. According to Fig 5B, dwell time towards the cave of the prior predictable outcome o1 seems to be higher than towards the caves of the prior less predictable outcomes. However, neither the main effect of outcome predictability, *F*(1,21) = 1.04, *p* = .320, nor its interaction with block, *F*(8,168)<1, reached significance. The main effect of block was significant, *F*(8,168) = 7.05, *p* < .001, *η*^*2*^_*p*_ = .251. None of the other main effects and interactions of interest were significant (largest *F* = 2.05, corresponding *p* = .154). A BF_01_ of 3.62 provided substantial evidence in favor of the models without the main effect of outcome predictability and decisive evidence for the models without the outcome predictability × block interaction (BF_01_ = 125). In line with Experiment 1, we additionally analyzed dwell time during the first half phase (Block 1 to 5), but the effect of outcome predictability did not reach significance, *F*(1,21)<1, *p* = .436.

In line with Experiment 2, the present experiment did not demonstrate an effect of the manipulation of the outcome predictability on subsequent learning. Thus, it seems that the results of Experiment 1 are not robust. Considering that the outcome predictability effect can be reliably demonstrated in other studies using different paradigms and designs [12–15], it is possible that the present design (Design 1) might not be conducive to observing the effect. We consistently observed that participants generally favored the outcome present in the middle cave (see S1 File). Although the duration of cue- and outcome-presentation, which was determined by a pilot study, ensures that participants cannot look at three caves in turn within one second (i.e. outcome-presentation) or detect the cave areas in peripheral vision when focusing on the fishing task, it is possible that they look at the middle cave and detect the other two caves in peripheral vision. Since the middle cave was the location of a less predictable outcome for the majority (two thirds) of participants, a robust demonstration of the outcome predictability effect might be impaired. Thus, we further assessed two different designs in the next experiments, presenting only two outcomes and no middle cave in both Phase 1 and Phase 2, to determine whether or not the outcome predictability effect can be reliably produced in the present paradigm.

## Experiment 4

According to the first three experiments, it seems that the manipulation of outcome predictability with Design 1 cannot reliably demonstrate an effect on subsequent learning. Thus, we developed two further designs (Design 2 and 3, see also Table 1), each degrading the contingency between cue C and outcome o2 in a different way in Phase 1 (Design 2 for Group *Outcome-absent*: A→o1, C→o2, C→Ø; Design 3 for Group *Cue-absent*: A→o1, A→o1, C→o2, Ø→o2) and examined their potential influences on Phase 2 learning (for both groups: X→o1, Y→o2, Z→Ø).

In addition, since the less predictable outcome o2 was presented *without* any cue half of the time in Phase 1 in Design 3 (Cue-absent condition), the contextual stimuli may provide some information about the imminent presentation of o2 and, thus, should be associated with o2 across Phase 1 training. Considering that the context in Phase 2 remained the same as in Phase 1, the context-o2 association formed in Phase 1 may transfer to Phase 2 learning and preferentially block learning about the novel cue associated with o2, as described above. In contrast, in Design 2 (Outcome-absent), although cue C was less predictive than cue A, it is still more informative than the contextual cue to predict o2 and, thus, can more effectively inhibit an association between the context and o2. In this manner, Phase 2 learning in Design 2 should be less likely to be influenced by any contextual association formed in Phase 1. Based on these considerations, if the outcome predictability effect is due to a change in the outcome’s associability, we expect an outcome predictability effect to occur in the outcome-absent Design 2, in particular. In contrast, if the effect relies on an influence of context associations rather than a change in the outcome’s associability, the effect should be observed in the cue-absent Design 3.

### Methods

#### Participants

Through an a priori power analysis, a sample size of 16 was suggested by G*Power [35] to be sufficient for Design 2 and 3 (*F* = 4.60) to detect a medium size effect of outcome predictability (Cohen’s f = .39 [36]) with 80% power (α = .05, two conditions for the within-subjects factor outcome predictability, two groups, correlation between repeated measures = .5 as default, no sphericity correction). Sixty-four undergraduate students from the Philipps-Universität Marburg, Germany (45 females, 19 males; M_age_ = 22.84 years, age range 19–47 years; 32 in each group) actually participated in the experiment. Data from seven additional participants were excluded from analysis.

#### Apparatus and stimuli

Only two outcomes (and their respective caves) were presented to participants during both training phases. The positions of two outcomes were counterbalanced, resulting in two cave conditions (o1 either in the top or the bottom cave). The cues within one learning phase were from the same modalities and the order of the two modalities (auditory vs. visual) was counterbalanced.

#### Design and procedure

Participants in the *Outcome-absent* group completed 96 trials grouped into 24 blocks (A→o1, B→Ø, C→o2, C→Ø) in Phase 1 and 36 trials grouped into 12 blocks (X→o1, Y→o2, Z→Ø) in Phase 2. For the *Cue-absent* group, Phase 1 training contained 90 trials grouped into 15 blocks (A→o1, A→o1, B→ Ø, B→ Ø, C→o2, Ø→o2) and 36 trials were arranged into 12 blocks in Phase 2 (X→o1, Y→o2, Z→Ø). The trial order was randomized within every three blocks. However, the sequence of outcome presentation during the first two trials in Phase 2 was counterbalanced.

### Results and discussion

#### Phase 1

**Fig 6:**
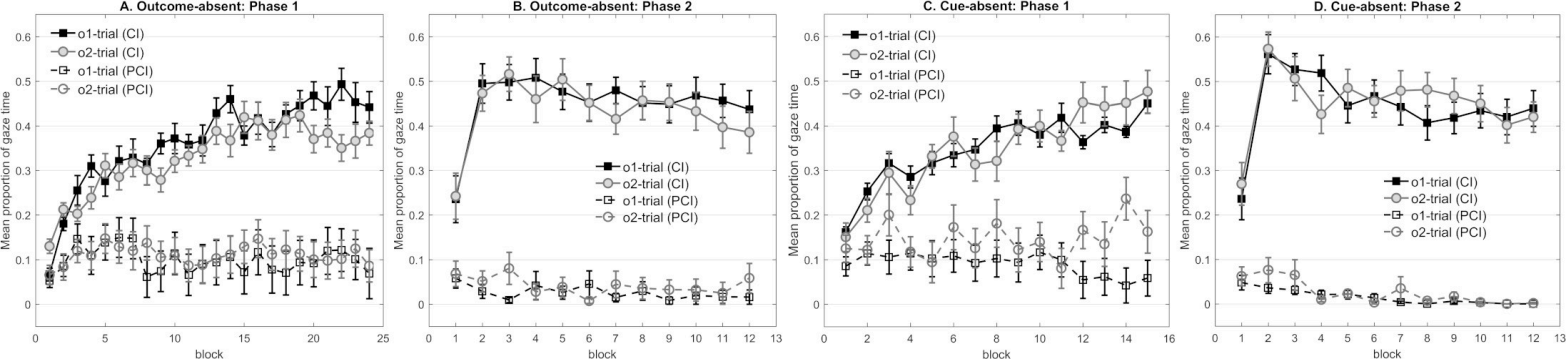
**Panel A to D represent the mean proportion of gaze time in Experiment 4 that participants looked at the correct outcome’s cave during the cue (CI) and the pre-cue (PCI) interval respectively in each phase.** (A) Mean gaze time of Group Outcome-absent across the 24 blocks in Phase 1. (B) Mean gaze time of Group Outcome-absent across the 12 blocks in Phase 2. (C) Mean gaze time of Group Cue-absent across the 15 blocks in Phase 1. (D) Mean gaze time of Group Cue-absent across the 12 blocks in Phase 2. Note that i) gaze time of Group Outcome-absent in responses to cue C in Phase 1 was averaged within each block, and ii) gaze behavior of Group Cue-outcome on the trials without signaling (Ø→o2) in Phase 1 was not presented in the figure.

Panel A and C of Fig 6 display the dwell times in Phase 1 for Group Outcome-absent and Cue-absent, respectively.

Statistical analyses were conducted for each group separately. For Group Outcome-absent data, responses to cue C were averaged across the two trials within each block (C→o2 and C→Ø) and referred to as o2-trial. As participants of Group Cue-absent could not respond to a cue during the Ø→o2 trial, those trials were not entered into analyses. Two 2 (outcome) × 2 (cave condition) × 12 (block) mixed design ANOVAs revealed that both groups demonstrated longer dwell times in anticipation of the predictable outcome o1 as compared to the less predictable outcome o2 (Group Outcome-absent: *F*(1,30) = 8.97, *p* = .005, *η*^*2*^_*p*_ = .230, Group Cue-absent: *F*(1,30) = 11.00, *p* = .002, *η*^*2*^_*p*_ = .268). We also found a significant main effect of block in both groups (Group Outcome-absent: *F*(23,690) = 7.04, *p* < .001, *η*^*2*^_*p*_ = .190, Group Cue-absent: *F*(14,420) = 8.49, *p* < .001, *η*^*2*^_*p*_ = .220). No other main factors or interactions of interest reached significance (the largest *F*- and the corresponding *p*-value across both groups: *F* = 1.31, *p* = .261).

#### Phase 2

Dwell time between two groups was compared to determine if Phase 2 learning differed due to different manipulations of outcome predictability (Fig 6, Panel B and D). A 2 (outcome predictability) × 2 (group) × 2 (cave condition) × 12 (block) mixed design ANOVA revealed a non-significant difference in dwell time towards the o1 area and towards the o2 area across groups, *F*(1,60) = 3.21, *p* = .078. None of the other main effects and interactions reached significance (largest *F* = 3.80, corresponding *p* = .056). When analyzing dwell time for each group in two ANOVAs with the factors outcome predictability, cave condition and block separately, Group Outcome-absent demonstrated a significant main effect of outcome predictability, *F*(1,30) = 6.66, *p* = .015, *η*^*2*^_*p*_ = .182. We also found a significant main effect of block in both groups (Group Outcome-absent: *F*(11,330) = 7.88, *p* < .001, *η*^*2*^_*p*_ = .208; Group Cue-absent: *F*(11,330) = 8.53, *p* < .001, *η*^*2*^_*p*_ = .221). No further main effects or interactions reached significance (the largest *F*- and *p*-value across both groups: *F* = 3.53, *p* = .070). Moreover, strong evidence in favor of the models without the main effect of outcome predictability was shown in Group Cue-absent (BF_01_ = 12.2), as well as decisive evidence for the models without the outcome × block interaction (BF_01_ = 250).

In the present experiment, both groups successfully discriminated the different predictability between two outcomes in Phase 1. Further analyses examining gaze behavior during the cue and the pre-cue interval separately (see S1 File) showed that presenting the outcome without a cue in half of the trials in Group Cue-absent (Design 3) increased dwell time towards the location of o2 during the pre-cue interval. Given that the context was consistently presented throughout the experiment, this may be regarded as a learned response to the context.

During Phase 2, Group Outcome-absent demonstrated better learning about the novel cue associated with the prior predictable outcome o1 compared to the prior less predictable outcome o2, while Group Cue-absent showed no difference in learning between the two trial types. Although Group Outcome-absent showed an outcome predictability effect, the additional analyses found that the effect observed in this group was based on longer gaze time towards the area of o2 than o1 during the pre-cue interval only. One possible interpretation is that participants were more strongly motivated to gaze at o2 in Phase 2 when they had experienced that o2 was less likely to be predicted in Phase 1. But if so, it is unclear why this bias was only evident during the pre-cue interval and why such an attentional preference did not benefit learning about the novel relationship with o2 in Phase 2. Another possible interpretation is that this bias indicates a stronger association between the context and o2 which blocked learning of a novel association with o2. However, we did not observe stronger contextual association with o2 in Phase 1, which would be evident by gaze behavior in the pre-cue interval in Phase 1. Thus, it remains open why such a strong context-o2 association suddenly manifested in Phase 2 training. Based on those considerations, the results of Group Outcome-absent require further replication (Experiment 5).

For Group Cue-absent, the additional analyses suggest that participants did not show preferential gazing towards the o2 area during the pre-cue interval in Phase 2, even though this was evident in Phase 1. One possibility of the missing effect is that Phase 2 learning reached asymptote too fast, as responses for both trial types reached the peak in Block 2. Moreover, it has been shown in the literature that the boundaries between phases is one factor of many failures to obtain other learning effects that rely on transfer of knowledge between two phases, for example, the *Kamin blocking* effect with human subjects [45]. If outcome predictability effects can be caused by transfer of contextual associations between phases, we should see greater effects if the experimental manipulation encourages this transfer (Experiment 6).

## Experiment 5

Having observed an effect of outcome predictability with Design 2 (A→**o1**, C→o2, C→Ø) on subsequent learning in Group Outcome-absent of Experiment 4, the present experiment aimed to replicate the effect and examined the potential influence of cognitive control on the outcome predictability effect. Given that some studies suggest that the learned predictiveness effect can be affected by manipulating participants’ beliefs about cue predictiveness between phases [46–48], we applied an instructional manipulation. For the *Continuity* group, participants were informed prior to Phase 2 that the predictable outcomes shown in Phase 1 were very likely to also be predictable in Phase 2. Since the instruction is consistent with the manipulation of predictability, it should encourage an outcome predictability effect. In contrast, the *Reversal* group were informed that the predictable outcomes shown in Phase 1 were unlikely to be predictable in Phase 2, which contradicted the actual design used in the experiment. Thus, we expected an observation of the outcome predictability effect in Group Continuity and a difference in Phase 2 learning between groups, if the effect is under cognitive control.

### Methods

#### Participants

Sixty-four undergraduate students from Philipps-Universität Marburg, Germany participated in this experiment (41 females, 23 males, *M*_age_ = 24.92 years, age range 19–39 years, 32 participants in each group). Data from three additional participants were excluded from analysis.

#### Design and procedure

The stimuli in the present experiment and design were the same as for Group Outcome-absent of Experiment 4. The additional instructions were presented prior to Phase 2 learning: Participants in Group Continuity were told that it was *very likely* that the awakening of the animal which had been predictable, would be predictable in the following phase. In contrast, Group Reversal received instructions that it was *very unlikely* that the previous predictable animal would be predictable in the next phase.

### Results and discussion

#### Phase 1

**Fig 7:**
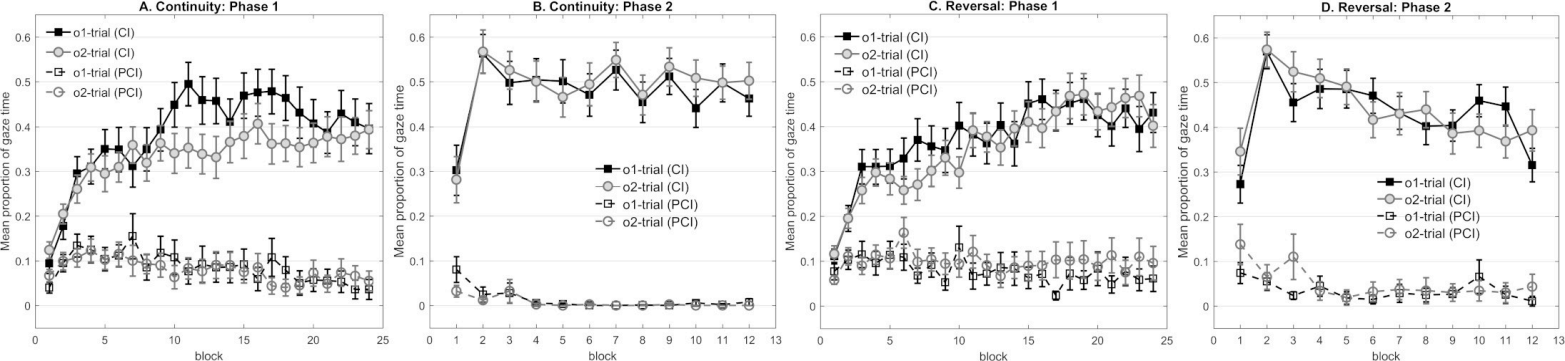
**Panel A to D represent the mean proportion of gaze time in Experiment 5 that participants looked at the correct outcome’s cave during the cue (CI) and the pre-cue interval (PCI) respectively in each phase.** (A) Mean gaze time of Group Continuity across the 24 blocks in Phase 1. (B) Mean gaze time of Group Continuity across the 12 blocks in Phase 2. (C) Mean gaze time of Group Reversal across the 24 blocks in Phase 1. (D) Mean gaze time of Group Reversal across the 12 blocks in Phase 2. Note that gaze time of both groups in responses to cue C in Phase 1 was averaged within each block.

In order to examine if each group had successfully learned the discrimination, two 2 (outcome) × 2 (cave condition) × 12 (block) mixed design ANOVAs were conducted. In line with the illustrations of Fig 7A and 7C, only Group Continuity showed longer dwell time in anticipation of o1 than o2 across blocks (main effect of outcome: *F*(1,29) = 5.39, *p* = .027, *η*^*2*^_*p*_ = .357; outcome × block interaction, *F*(23,667) = 1.94, *p* = .032, *η*^*2*^_*p*_ = .063). A significant main effect of block was shown in both groups (Continuity: *F*(23,667) = 9.24, *p* < .001, *η*^*2*^_*p*_ = .242, Reversal: *F*(23,690) = 10.36, *p* < .001, *η*^*2*^_*p*_ = .257). None of the other main effects or interactions reached significance (the largest *F*- and the corresponding *p*-value across both groups: *F* = 2.62, *p* = .116).

#### Phase 2

A comparison of two groups was estimated with a 2 (outcome predictability) × 2 (group) × 2 (cave condition) × 12 (block) mixed design ANOVA. The main analysis did not show any difference in Phase 2 learning between groups based on the prior predictability of outcomes. We found a significant main effect of block, *F*(11,660) = 19.24, *p* < .001, *η*^*2*^_*p*_ = .243, and a significant group × block interaction, *F*(11,660) = 2.11, *p* = .039, *η*^*2*^_*p*_ = .034. No other main effects or interactions of interest reached significance (largest *F* = 3.80, corresponding *p* = .056). Further, we found strong evidence in favor of the models without the main effect of outcome predictability (BF_01_ = 15.38), anecdotal evidence for the models without the outcome predictability × group interaction (BF_01_ = 1.91), and decisive evidence for the models without the outcome predictability × block interaction (BF_01_ = 4222.97). Moreover, decisive evidence was shown in favor of the models without the three-way interaction (BF_01_ = 500).

In the present experiment, only Group Continuity successfully discriminated the difference in the outcomes’ predictability in Phase 1 and, in line with our expectation, the difference was based on stronger responses to the cue associated with the predictable outcome o1 as compared to the cue with the less predictable outcome o2 during the cue interval (see analyses in S1 File). However, participants in Group Continuity did not demonstrate the outcome predictability effect, even though participants were explicitly informed about the outcome’s predictability prior to Phase 2 training. The results suggest that the effect observed in Experiment 4 is either unreliable, or is so fragile that merely highlighting the presence of these relationships causes the effect to disappear. As a point of contrast, the learned predictiveness effect is known to be substantially more robust after similar explicit instructions [e.g. 46–48].

For Group Reversal, contrary to our expectation, learning about the predictable and the less predictable outcome in Phase 1 did not differ from each other, even though both groups were treated identically in Phase 1. Because of the results of Phase 1 learning, it is impossible to determine whether the failure to observe the outcome predictability effect in Phase 2 was due to the unsuccessful discrimination of the outcome’s predictability in Phase 1 or the instructional manipulation.

## Experiment 6

Experiment 6 returned to the Cue-absent condition used in Experiment 4 to further explore the possibility of context blocking. In this group in Experiment 4, implementing a stronger contextual association with the less predictable outcome o2 formed in Phase 1 (Design 3: A→o1, A→o1, C→o2, Ø→o2) did not actually block learning about o2’s novel relationship in Phase 2. We hypothesized that the boundaries between phases may prevent the blocking effect [45]. Thus, the present experiment aimed to enhance the transfer of strong contextual associations learned in Phase 1, to determine whether these associations will produce an outcome predictability effect in Phase 2. For this purpose, we removed the break between two phases. Moreover, we included an additional transit block (A-o1 and Ø-o2) between the phases to remind participants of the different predictability of o1 and o2 shortly prior to Phase 2 training.

### Methods

#### Participants

Thirty-two undergraduate students from the University of Sydney (25 females, 7 males; M_age_ = 18.23 years, age range 17–22 years) participated in this experiment. All participants received a complete description of the experiment and were asked to sign a written informed consent prior to data collection. Data from five additional participants were excluded from analysis.

#### Stimuli, design and procedure

The stimuli and design were the same as Experiment 4 with three exceptions. First, the modality of cues was not counterbalanced to reduce the number of the counterbalancing conditions: Auditory cues were presented in Phase 1 and visual cues in Phase 2. Second, the X-o1 trial was introduced on the first trial of Phase 2 for half of the participants while the Y-o2 trial for the other half. Third, Phase 1 training contained 84 trials grouped into 14 blocks (A→o1, A→o1, B→ Ø, B→Ø, C→o2, Ø →o2) and two additional trials as Block 15 (A→o1, Ø→o2). In Phase 2, 60 trials were arranged into 10 blocks (X→o1, Y→o2, Z→Ø, R→o1, S→o2, T→Ø). The trial order was randomized within every two blocks. The order of two trials in Block 15 was counterbalanced. A short break was introduced after the first ten blocks in Phase 1 and a drift check was performed after Phase 2 training.

### Results and discussion

#### Phase 1

**Fig 8:**
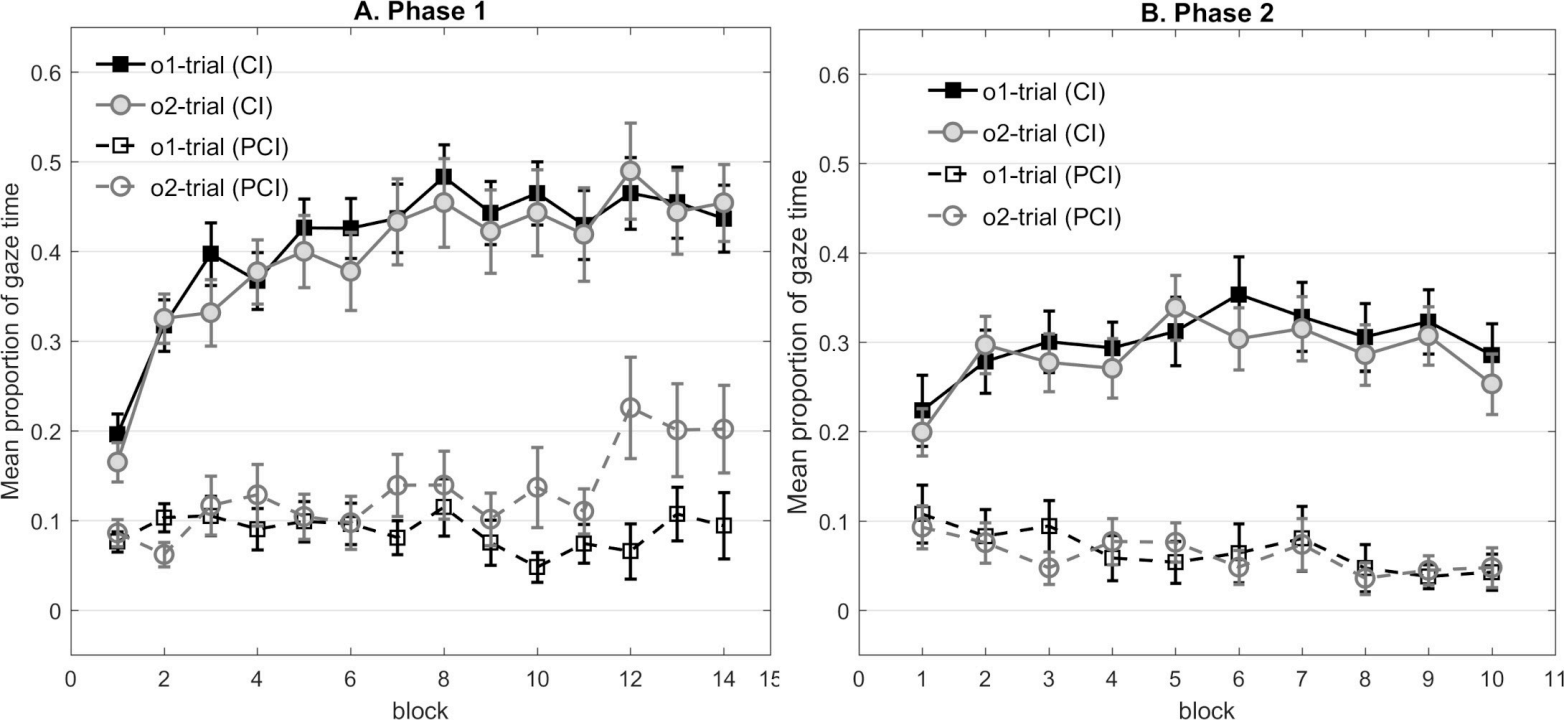
Panel A and B represent the mean proportion of gaze time in Experiment 6 that participants looked at the correct outcome’s cave during the cue (CI) and the pre-cue interval (PCI) respectively in each phase. (A) Mean gaze time across the 14 blocks in Phase 1. (B) Mean gaze time across the ten blocks in Phase 2. Note that i) gaze behavior on the trials without signaling (Ø→o2) in Phase 1 was not presented in the figure, and ii) gaze time in Phase 2 was averaged based on the predictability of each trial’s outcome in Phase 1.

Fig 8A displays dwell times during Phase 1. Since participants could not respond to a cue during the Ø→o2 trials, the gaze behavior of these trials could not be analyzed. A 2 (outcome) × 2 (cave condition) × 14 (block) mixed design ANOVA was conducted. The main analysis yielded a significant main effect of outcome, *F*(1,29) = 9.37, *p* = .005, *η*^*2*^_*p*_ = .244, a main effect of block, *F*(13,377) = 6.20, *p* < .001, *η*^*2*^_*p*_ = .176, as well as an interaction with cave condition. No further main effects or interactions of interest were significant (largest *F* = 2.73, corresponding *p* = .109).

#### Phase 2

Dwell time was averaged within each block based on the outcome’s predictability during Phase 1, resulting in two trial types (trials involving prior predictable outcomes vs. trials involving prior less predictable outcomes). A 2 (outcome predictability) × 2 (cave condition) × 10 (block) mixed design ANOVA was conducted. In line with Fig 8B, the main analysis did not reveal a difference in dwell time between the two trial types. We only observed a significant main effect of block, *F*(9,270) = 5.71, *p* < .001, *η*^*2*^_*p*_ = .160. No other main effects or interactions of interest reached significance (largest *F* = 3.27, corresponding *p* = .081). Moreover, substantial evidence was provided in favor of the models without the main effect of outcome predictability (BF_01_ = 7.69), as well as decisive evidence for the models without the predictability × block interaction (BF_01_ = 250).

In line with our expectations, participants showed slightly better learning about the predictable outcome o1 than the less predictable outcome o2 in Phase 1. Additional analyses also confirmed a preference for the o2 area over o1 during the pre-cue interval, indicating a stronger context-o2 association elicited across training (see S1 File). However, the manipulation of outcome predictability in Phase 1 did not bias learning about the novel relationships with o1 and o2 in Phase 2. In addition, we noted that the strong context-o2 association shown in Phase 1 did not manifest in Phase 2. Possibly, participants might immediately realize the new patterns would be present, once a novel cue appeared, and are therefore less likely to be influenced by their previous experience.

## General discussion and conclusion

In recent years, the question has been raised as to whether the associative history of outcomes plays a role in subsequent learning about novel cue-outcome associations, beyond its influence in calculations of prediction error [16 for a review, see also 49]. In six experiments, we manipulated outcome predictability using three different designs, and investigated whether this manipulation would bias subsequent learning about the outcomes, when each of them became fully predictable by novel cues (for a summary of the results in all experiments see Table 3). Although data from all experiments confirmed that each design mostly succeeded in distinguishing the outcomes’ predictability in the first training phase, we did not demonstrate a reliable outcome predictability effect in our goal-tracking task, as the effect was only observed in two of six experiments (i.e. Experiment 1 and the Outcome-absent group of Experiment 4).

**Table 3:** Summary of predictions and results.

Exp.	Group	Prediction	Results
1	-	Demonstration of OP effect with Design 1	Effect observation
2	NoShift-3cave	Replication of the effect observed in Exp.1	No observation
	NoShift-2cave	Effect replication with elimination of the interference of the middle cave preference	No observation
	Shift-3cave	Effect observation is expected, if it is context-independent.	No observation
	Shift-2cave	Effect observation with a manipulation to eliminate the middle cave preference is expected, if it is context-independent.	No observation
3	-	Replication of Exp.1	No observation
4	Outcome-absent	demonstration of OP effect with Design 2	Effect observation
	Cue-absent	demonstration of OP effect with Design 3	No observation
5	Continuity	Replication of the effect observed in Group Outcome-absent (Design 2) of Exp.4 with an instructional manipulation	No observation
	Reversal	Demonstration of OP effect with a reversed instructional manipulation is expected, if it is not affected by a higher cognitive control.	No observation
6	-	Demonstration of OP effect with Design 3 by applying a manipulation to strengthen the transfer of contextual associations between phases	No observation

*Note*. OP effect is the abbreviation of the outcome predictability effect.

Considering the nonsignificant findings in most of the experiments, the first concern is whether the present study is under-powered. We discuss three different approaches to answer this question: A priori analyses of sample size and power, confidence intervals for the estimated population effect sizes, and Bayes factors. For analyses of sample sizes, results from the study by Griffiths et al (2015) were considered for the estimations of effect size, as at that time of the first data collection the outcome predictability effect had been only reported by this study [13]. As their observed effect sizes fell into a range from medium to large, we assumed a medium size effect of outcome predictability (Cohen’s f = .39 [36]) to be observed. However, we need to note the limitation of basing an effect size on a single study and, further, the distinction between our procedure and that used by Griffiths and colleagues [13, see also below]. Taking consideration of publication bias [50–52], it is arguable that the true effect size might be smaller than a medium size that we initially assumed. Moreover, the a priori analyses of power was done using a simplified design as the estimation of power for complex mixed design ANOVAs is non-trivial [53, 54]. Most power and/or sample size methods, e.g., G*Power, have been restricted to those designs involving a single within-subjects variable and two-way mixed designs [35]. In tools that are available for two-factor repeated measure design, the number of levels for each factor is restricted [e.g., 55]. Therefore, and in addition to justified questions about the appropriate estimate of the effect size as well as assumptions about the correlations between repeated measures, the results of our initial analyses of sample sizes and power have to be interpreted cautiously. Calculating confidence intervals around the estimated effect, *η*^*2*^_*p*_, can help determine whether a nonsignificant result indicates the true absence of an effect rather than a lack of power [56], as the true value of the population effect lies within this interval [57]. The lower bound for most of the experiments includes values of zero and therefore no effect at all (Table 4, [58, 59]), which is in line with null hypothesis significance testing of the ANOVAs. However, the confidence intervals also indicate a rather low precision of the estimate of plausible values for the population effect, with the upper bound including medium effects for most of the experiments. The strongest support for a true absence of effects therefore stems from the Bayesian statistics [40]. The Bayes factors provided substantial evidence [42] for the null hypothesis in the conditions that did not demonstrate the outcome predictability effect. Considering all different approaches and results across the six experiments, we would argue that, even though we cannot rule out the possibility that the studies were underpowered to detect the effect, it is unlikely that a simple lack power can account for the repeatedly non-significant results in the present study.

**Table 4:** Observed effect size and confidence interval.

	Exp1.		Exp.2	Exp3	Combi. Exp1&3	Exp4		Exp5	Exp6
	nine blocks	five blocks	NoShift-3cave	-		Outcome-absent	Cue-Absent	Conti-nuity	-
*η*^*2*^_*p*_	.164	.217*	.008	.047	.103*	.182*	.0004	.031	.007
*CI*	[0, .377]	[.016, .427]	[0, .148]	[0, .237]	[.004, .255]	[.021, .366]	[0, .016]	[0, .178]	[0, .117]

*Note*. Effect sizes were represented by the partial eta squared for the main effect of outcome predictability in Phase 2 learning. CI denotes 90% confidence interval, corresponding to α = .05, on the effect size *η*^*2*^_*p*_ [58]. For Exp. 2 and 5 with a multi-group design, only the replication group was reported. Combi. Exp.1&3 represents combined data set of Experiment 1 and 3, and the results reported in the table were based on the analyses of dwell time across all nine blocks in Phase 2.

*: the main effect of outcome predictability was significant in the experiment (*p* < .05).

A general preference to gaze at the middle cave, which was more often the location of the less predictable outcome, may have worked against the observation of an outcome predictability effect in Experiment 2 and 3. However, the effect was also not reliably observed when this factor was removed in Experiment 4–6. It is therefore unlikely that this is the only factor relating to our failure to demonstrate the outcome predictability effect.

Another notable result of the present study is the failed demonstration of the outcome predictability effect with Design 3 (Cue-absent). The idea that inducing a contextual association with the less predictable outcome could block subsequent learning with that outcome, can be related to the so-called US pre-exposure effect [60–62]. When subjects are exposed to an outcome alone prior to cue-outcome pairing (i.e. the outcome is experienced as less predictable), subsequent learning about the cue-outcome relationship is impaired. Based on an associative account, the common explanation of the effect is that an association between the outcome and contextual stimuli formed during the pre-exposure phase blocks learning about the relationship between a novel cue and this outcome [63–66]. Although the context blocking effect is thought to be relatively robust, we did not find the effect in the present study. In particular, Experiment 6 successfully induced strong contextual associations in Phase 1, but still failed to exert any effect on learning in Phase 2. One possibility is that the outcome predictability effect does not rely on a blocking effect caused by context. However, an alternative is that transfer of contextual associations between phases is not established in the present paradigm. It appears that participants are specifically sensitive to a change in pattern [67], such as the sudden presentation of novel stimuli or a change in the cue-outcome contingencies. As soon as they notice the new pattern present in Phase 2, the experience from the prior phase would be ignored.

Other demonstrations of the outcome predictability effect have now been reported in four articles using three different protocols [12–15]. The effect was produced using a human causal learning allergist task [12, 15], a visual cued search task [13] and a serial letter-prediction task [14]. Considering the diverse designs and methods used in these studies compared to ours, careful consideration of the data from all related studies is essential to detect contributing factors in our unreliable observation of the effect.

Comparing the procedure used in the present experiment with other related studies, a distinction is evident between our study and Griffiths et al., which uses a causal learning allergist task [13]. Participants in our study were not required to make an explicit outcome prediction and were only confronted with one outcome in each trial. In comparison, their study presented outcomes with different (previous) predictability in compound and asked participants to recall explicit causal knowledge regarding the cues and the outcomes. In this way, participants might experience greater subjective discrepancy in the outcome’s predictability than would be the case with the single outcome presentation used in this study. Moreover, we analyzed data from accurate responses *during* the process of learning. In contrast, their main findings were observed via likelihood rating in a distinct test of explicit knowledge that occurred only once learning was complete. In this manner, their critical data represent a bias in causal reasoning during the test phase, while measures in the present study captured changes in accurate responses to represent changes in associative formation during learning. Hence, it seems that two paradigms measure learning and performance in distinctly different ways, possibly contributing to the difference in observations of the effect in these studies.

However, other existing studies about the outcome predictability effect used a paradigm more similar to the current goal-tracking task [12, 14], and have confirmed that the effect occurs using a single-outcome design and capturing responses *during* learning. Therefore, none of these factors can be the sole reason for the current failure to observe the effect. However, there are still several methodological differences. Perhaps the most obvious one concerns the dependent variable as both Griffiths et al. [12] and Quigley, et al. [14] measured response latency to indicate learning. It might simply be the case that the effect is only observed during training when participants are put under time pressure to respond. In the current paradigm, participants performed more efficiently if they not only learned which cue predicted which outcome but also to delay responding (depending on how fast they could move the mouse from the rive to the cave) as the outcome would only appear 3.66 to 4.66 sec after the cue onset.

In the study using the visual cued search task by Griffiths et al. [12], participants were asked to respond as soon as possible based on the orientation of a presented arrow which could occur in one of eight locations. They manipulated the locations (i.e. outcome) of the array present in Phase 1 to indicate whether it can be perfectly predicted by a given cue (for Experiment 2: A→**o1**, B→**o2**, C→**o3/o4**, D→**o3/o4**, E→**o1/o2**/…/o8). In Phase 2, the novel cues were all fully predictive of the locations of the arrow (F→**o1**, G→**o2**, H→**o3**, I→**o4**, J→**o1/o2**/…/o8, invalid cue E in Phase 1 and cue J in Phase 2 preceded the target appearing equally often in all of the 8 possible locations). Therefore, participants experienced a wider range of outcome predictability and cue predictiveness in both phases of this design compared to the design in the current study, where only two amounts of predictability and predictiveness were used in Phase 1 and all outcomes were predictable in Phase 2. In contrast to our data, they demonstrated a reliable effect of outcome predictability on Phase 2 learning. Interestingly, this effect depended on the cue as they observed faster responses for prior predictable than less predictable outcome locations only following a *valid* cue (e.g., F→**o1**) whereas responses was faster for prior less predictable than predictable locations following the *invalid* cue (e.g. J→**o3**). Such an observation is not completely consistent with an expectation based on Mackintosh-like process. If the associability of o1 became greater than o3 after Phase 1, it should not only be more readily associated with F but also with J. Instead, Griffiths et al. [12, 13, see 16 for a review] suggested that participants may establish a model which contains the information about the degree of cue predictiveness and outcome predictability in Phase 1 [68 for a view of causal model, see also 69], and then in Phase 2 preferentially link the cues with outcomes which match in this manner (i.e. linking the previous predictable outcome to the predictive cues and the previous less predictable outcome to the non-predictive cue). If this explanation of the results is correct, the fact that participants experienced only two kinds of predictiveness and predictability in Phase 1 and only predictable outcomes in Phase 2 might reduce the motivation or ability to both establish a model of predictability in Phase 1 and use it to match it to the new cues in Phase 2. Relating to the present study, since we did not provide an additional less predictable outcome in Phase 2 to be associated with novel cues, it is unnecessary for participants to exhaust all learned experience in the past, including previous predictability of outcomes, to find a better match.

In the study by Quigley, et al. [14], participants were required to learn about the relationships between letters, and correspondingly press target buttons X and Z. In a modified design for Experiment 2, outcome X and Z differed in their predictability in Phase 1 (e.g., P→**X**, F/G/W→**Z**/G/W). Also, the two letters S and H that served as cues to predict outcome X and Z in Phase 2 (e.g., H→**X**, S→**Z**), were already present in Phase 1 (H→S, S→H). In a final test phase, participants were required to give likelihood rating for the target stimuli. An effect of outcome predictability was observed in Phase 2 learning as well as in the test phase. Notably, all Phase 1 pairings were also presented in Phase 2 (e.g., P→**X**, F/G/W→**Z**/G/W), except the pairings between H and S. We note that these manipulations might be critical to for demonstrating the outcome predictability effect in their study. At the very least, they invite other interpretations of the results besides an indication of change in *β* suggested by the authors. First, if a stronger contextual association was elicited with the less predictable outcome Z in Phase 1, the context-Z association might be transferred to Phase 2 and block learning about the novel S-Z relationship. Second, although Z is fully predictable to when S occurs in Phase 2, it is still less predictable than X in a general manner considering all other Z-pairings present in Phase 2 (e.g., F/G/W→**Z**/G/W). Consequently, the difference in responding for X and Z could rely on either previous or current predictability, the latter of which could be due to well-established contingency learning mechanisms. Any difference in learning about X and Z that is due to differences in their predictability in Phase 2 does not necessitate *any* conclusion about a change in their processing as a consequence of learning their predictabilities in Phase 1. Third, given that H and S were consistently paired with each other in Phase 1, their processing may change as a consequence of always being either predictive or predictable (for instance S might be encoded as being “good for prediction” because of a combination of its predictiveness and predictability). In Phase 2, if Z was encoded as “less predictable” relative to all stimuli, then S might be less readily linked to Z as they did not match each other in their predictive properties. This account is in line with the arguments about linking cues and outcomes based on their (previous) predictiveness and predictability proposed by Griffiths et al. [12, 13]. In summary, even though their experiments investigated the same research question, their very specific manipulations make it difficult to conclude whether we should have expected the same effects in the present study.

Overall, data from the six experiments in the present study did not reliably demonstrate an effect of outcome predictability on subsequent learning in our goal-tracking paradigm. We have reviewed all existing related studies [12–14] and found methodological distinctions that may contribute to differences in the effect. This leads to several observations that need to be investigated in future research. (1) The outcome predictability effect is not reliably demonstrated in a human conditioning paradigm with goal-tracking. If this turns out to be true of other conditioning-like paradigms, it suggests the outcome predictability effect is not generalizable in the same way as other related and widely observed phenomena such as the learned predictiveness effect. (2) The effect observed in different paradigms might not have a single cause. It is unclear at this point whether the existing demonstrations are all caused by a change in the associability of the outcome, for instance. Further investigations are required to gain greater understanding of the effect and the role of outcome-processing in associative learning.

## Supporting information

S1 File (DOCX)
