# Supplementary material for: Does learning history shape the associability of outcomes? Further tests of the outcome predictability effect
Source: PLoS One. 2020 Dec 18;15(12):e0243434. doi: 10.1371/journal.pone.0243434 (PMC7748133; doi:10.1371/journal.pone.0243434)
Supplement: S1 File — (DOCX) [file pone.0243434.s001.docx]

The counterbalancing factor “cave condition” was included in ANOVAs for each experiment in the main text, in order to reduce error variance. As its effects and interactions are not of interest for the research question, the corresponding statistical results are reported in the supplementary material. Further, additional analyses were conducted for each experiment, in which gaze time during the cue and the pre-cue interval (CI and PCI) were analyzed separately. Results of those additional analyses were also reported in the supplementary material.

# Experiment 1

Phase 1.

The main analysis, using a 3 (outcome) × 3 (cave condition) × 12 (block) ANOVA with within-subjects factors of outcome and block, revealed a significant outcome × cave condition interaction, *F*(4,42)=4.3, *p*=.005, *η^2^_p_*=.291, suggesting that participants looked at the middle cave generally longer than at the other two.

The additional analyses using the analog ANOVAs examined gaze time during the CI and the PCI separately. During the CI, the main effect of outcome, *F*(2,42)=11.73, *p*<.001, *η^2^_p_*=.359, the main effect of block, *F*(11,231)=3.55, *p*=.003, *η^2^_p_*=.145, and their interaction, *F*(22,462)=2.71, *p*<.001, *η^2^_p_*=.114, were significant. In addition, we also observed a significant outcome × cave condition interaction, *F*(4,42)=8.91, *p*<.001, *η^2^_p_*=.459. None of the other main effects and interactions during the CI and the PCI were significant (largest *F*=2.46, corresponding *p*=.060).

Phase 2.

The additional analyses examined gaze time across 9 blocks during the CI and the PCI respectively, using an 2 (outcome predictability) × 3 (cave condition) × 9 (block) ANOVA with within-subjects factors of outcome predictability and block. During the CI, we observed a significant main effect of outcome predictability, *F*(1,21)=4.41, *p*=.048, *η^2^_p_*=.173, and a significant main effect of block, *F*(8,168)=5.78, *p*<.001, *η^2^_p_*=.216. None of the other main effects and interactions during the CI and the PCI were significant (largest *F*=1.35, corresponding *p*=.225).

# Experiment 2

Phase 1.

The main analysis was conducted for each group, using a 3 (outcome) × 3 (cave condition) × 12 (block) ANOVA with within-subjects factors of outcome and block. The counterbalancing factor cave condition significantly interacted with other factors in all four groups (outcome × cave condition interaction for NoShift-3cave: *F*(4,42)=9.84, *p*<.001, *η^2^_p_*=.484, for NoShift-2cave: *F*(1,22)=6.36, *p*=.019, *η^2^_p_*=.224, for Shift-3cave: *F*(4,42)=9.91, *p*<.001, *η^2^_p_*=.486 and for Shift-2cave: *F*(1,22)=10.84, *p*=.003, *η^2^_p_*=.330; cave condition × block interaction for NoShift-2cave: *F*(11,242)=3.11, *p*=.018, *η^2^_p_*=.124).

The additional analyses using an analog ANOVA examined gaze time during the CI and the PCI for each group, respectively. The results were similar to those of the main analyses: In all four groups, we observed the significant main effect of outcome (NoShift-3cave: *F*(2,42)=9.13, *p*=.001, *η^2^_p_*=.303; NoShift-2cave: *F*(1,22)=10.91, *p*<.001, *η^2^_p_*=.342; Shift-3cave: *F*(2,42)=27.67, *p*<.001, *η^2^_p_*=.557; Shift-2cave: *F*(1,22)=35.01, *p*<.001, *η^2^_p_*=.614), and its interaction with block (NoShift-3cave: *F*(22,462)=2.26, *p*=.001, *η^2^_p_*=.097; NoShift-2cave: *F*(11,242)=2.09, *p*=.003, *η^2^_p_*=.091; Shift-3cave: *F*(22,462)=4.61, *p*<.001, *η^2^_p_*=.173; Shift-2cave: *F*(11,242)=1.87, *p*=.044, *η^2^_p_*=.078). The main effect of block was significant in Group NoShift-3cave, *F*(11,231)=4.18, *p*<.001, *η^2^_p_*=.166, Shift-3cave (*F*(11,231)=3.23, *p*=.007, *η^2^_p_*=.114, and Shift-2cave, *F*(11,242)=2.95, *p*=.010, *η^2^_p_*=.118. In addition, the cave condition significantly interacted with other factors (outcome × cave condition interaction for NoShift-3cave: *F*(4,42)=11.02, *p*<.001, *η^2^_p_*=.512, for NoShift-2cave: *F*(1,22)=10.20, *p*<.001, *η^2^_p_*=.493, for Shift-3cave: *F*(4,42)=6.80, *p*=.016, *η^2^_p_*=.236 and for Shift-2cave: *F*(1,22)=13.14, *p*=.001, *η^2^_p_*=.374; cave condition × block interaction for NoShift-2cave: *F*(11,242)=4.16, *p*=.002, *η^2^_p_*=.159). During the PCI, we observed the significant main effect of block in all four groups (NoShift-3cave: *F*(11,231)=4.22, *p*=.011, *η^2^_p_*=.167; Shift-3cave: *F*(11,231)=24.94, *p*<.001, *η^2^_p_*=.543; NoShift-2cave: *F*(11,242)=6.80, *p*=.002, *η^2^_p_*=.236; Shift-2cave: *F*(11,242)=15.961, *p*<.001, *η^2^_p_*=.420). Moreover, Shift-3cave showed a significantly lower gaze time towards o3 area than o1 and o2 area (*F*(2,42)=3.93, *p*=.027, *η^2^_p_*=.158), and a significant outcome × cave condition interaction *F*(4,42)=2.73, *p*=.048, *η^2^_p_*=.206). No further main effects or interactions in all analyses during the CI and the PCI reached significance (largest *F*=3.34, corresponding *p*=.081).

Phase 2.

The main analyses compared two pairs of groups (NoShift-3cave vs. Shift-3cave, NoShift-2cave vs. Shift-2cave), using a 2 (outcome predictability) × 2 (Group NoShift vs. Shift) × 3 (cave condition for 3cave) or 2 (cave condition for 2cave) × 12 (block) ANOVA with within-subjects factors of outcome predictability and block. We observed some significant interactions with the counterbalancing factor cave condition (Shift-3cave &. Shift-3cave: cave condition × outcome predictability interaction *F*(2,41)=16.09, *p*<.001, *η^2^_p_*=.440; NoShift-2cave &. Shift-2cave: cave condition × outcome predictability interaction *F*(1,44)=15.19, *p*<.001, *η^2^_p_*=.257, cave condition × outcome predictability × group interaction *F*(1,44)=5.36, *p*=.025, *η^2^_p_*=.109).

The additional analyses using an analog ANOVA examined gaze time during the CI and the PCI for each pair of groups, respectively. The additional analyses for the CI revealed a significant main effect of block (NoShift-3cave &. Shift-3cave: *F*(11,451)=6.70, *p*<.001, *η^2^_p_*=.138; NoShift-2cave &. Shift-2cave: *F*(11,484)=5.70, *p*<.001, *η^2^_p_*=.115). We also observed a significant cave condition × outcome predictability interaction for NoShift-3cave and Shift-3cave, *F*(2,41)=17.00, *p*<.001, *η^2^_p_*=.447, and for NoShift-2cave &. Shift-2cave, *F*(1,44)=17.94, *p*<.001, *η^2^_p_*=.290. For NoShift-2cave and Shift-2cave, a cave condition × outcome predictability × group interaction was significant, *F*(1,44)=6.47, *p*=.015, *η^2^_p_*=.128, indicating that Shift-2cave showed more preference for the top cave over the bottom cave than NoShift-2. For the PCI, the analyses only revealed a significant main effect block when comparing NoShift-2cave and Shift-2cave, *F*(11,484)=3.63, *p*=.027, *η^2^_p_*=.076. None of other main effects and interactions in all analyses during the CI and the PCI reached significance (largest *F* =3.08, corresponding *p*=.087).

# Experiment 3

Phase 1.

The main analysis, using a 3 (outcome) × 3 (cave condition) × 12 (block) ANOVA with within-subjects factors of outcome and block, revealed a significant cave condition × outcome interaction, *F*(4,42)=12.09, *p*<.001, *η^2^_p_*=.535.

The additional analyses using the analog ANOVAs examined gaze time during the CI and the PCI separately. The results for the CI are similar to those from the main analysis: We observed a significant main effect of outcome, *F*(2,42)=21.60, *p*<.001, *η^2^_p_*=.507, a significant outcome × block interaction: *F*(22,462)=2.72, *p*=.009, *η^2^_p_*=.115, and a significant main effect of block, *F*(11,231)=3.83, *p*=.007, *η^2^_p_*=.154. In addition, a cave condition × outcome interaction was significant, *F*(4,42)=12.24, *p*<.001, *η^2^_p_*=.538. For the PCI, we only observed a significant main effect of block, *F*(11,231)=7.81, *p*=.001, *η^2^_p_*=.271. None of the other main effects and interactions during the CI and the PCI were significant (largest *F*=1.59, corresponding *p*=.216).

Phase 2.

The main analysis, using a 2 (outcome predictability) × 3 (cave condition) × 9 (block) ANOVA with within-subjects factors of outcome and block, demonstrated a significant cave condition × outcome predictability interaction, *F*(2,21)=4.03, *p*=.033, *η^2^_p_*=.277, showing a general longer gazing towards the middle cave.

The additional analyses using the analog ANOVAs examined gaze time across 9 blocks during the CI and the PCI separately. We only observed a significant main effect of block, *F*(8,168)=6.80, *p*<.001, *η^2^_p_*=.245, and a significant cave condition × outcome predictability interaction for the CI, *F*(2,21)=4.12, *p*=.031, *η^2^_p_*=.282. None of the other main effects and interactions during the CI and the PCI were significant (largest *F*=3.05, corresponding *p*=.060).

# Combining Experiment 1 and 3

Since Experiment 3 was a direct replication of Experiment 1, we combined data of two experiments to conduct additional analyses with larger sample size.

Phase 1.

A 3 (outcome) × 2 (Experiment) × 3 (cave condition) × 12 (block) ANOVA within-subjects factors of outcome and block revealed a significant main effect of outcome, *F*(2,84)=26.36, *p*<.001, *η^2^_p_*=.386, with significant contrasts regarding the comparison between o1 and both o2 and o3 trials, but not o2 versus o3, *F*_o1vs.o2_=30.20, *p*<.001, *F*_o1vs.o3_=32.93, *p*<.001, *F*_o2vs.o3_=1.34, *p*=.253. Notably, a significant outcome × block interaction reflected that dwell time increased more rapidly in o1 trials than in o2 or o3 trials, *F*(22,924)=4.00, *p*<.001, *η^2^_p_*=.087. Further, the main effect of block was significant *F*(11,462)=7.16, *p*<.001, *η^2^_p_*=.146. Additionally, we also observed a significant outcome × cave condition interaction, *F*(4,84)=14.26, *p*<.001, *η^2^_p_*=.404, showing a general preference for the middle cave over the other two. None of the other main effects and interactions were significant (largest *F*=3.20, corresponding *p*=.081).

The additional analyses using the analog ANOVAs examined gaze time during the CI and the PCI separately. The results for the CI are similar to those from the main analysis: The main effect of outcome, *F*(2,84)=32.14, *p*<.001, *η^2^_p_*=.434, the main effect of block, *F*(11,462)=6.43, *p*<.001 *η^2^_p_*=.133, as well as the outcome × block interaction: *F*(22,924)=4.27, *p p*<.001, *η^2^_p_*=.092, were significant. In addition, a cave condition × outcome interaction was significant, *F*(4,84)=20.29, *p*<.001, *η^2^_p_*=.491. For the PCI, the analysis revealed a significant main effect of Experiment, *F*(1,42)=6.44, *p*=.015, *η^2^_p_*=.133, showing that participants from Experiment 1 spent overall longer time towards all outcome areas before the presence of cues than participants from Experiment 3. In addition, we observed a three-way cave condition × outcome × Experiment interaction, *F*(4,84)=2.48, *p*=.050, *η^2^_p_*=.106, suggesting participants from Experiment 1 more strongly preferred the middle cave over other two than those from Experiment 3 during the PCI. None of the other main effects and interactions during the CI and the PCI were significant (largest *F*=2.41, corresponding *p*=.055).

Phase 2.

A 2 (outcome predictability) × 2 (Experiment) × 3 (cave condition) × 9 (block) ANOVA with within-subjects factors of outcome predictability and block revealed a significant main effect of outcome predictability, *F*(1,42)=4.84, *p*=.033, *η^2^_p_*=.103, showing longer dwell time in anticipation of o1 than the other two outcomes in Phase 2. We also observed a significant main effect of block, *F*(8,336)=12.53, *p*<.001, *η^2^_p_*=.230. But the outcome × block interaction did not reach significance, *F*(8,336)=1.40, *p*=.217, *η^2^_p_*=.032. In addition, cave condition significantly interacted with outcome predictability, *F*(2,42)=3.57, *p*=.037, *η^2^_p_*=.145. None of the other main effects and interactions were significant (largest *F*=1.59, corresponding *p*=.094). In line with Experiment 1, we additionally analyzed dwell time during the first half phase (Block 1 to 5). As results, an significant main effect of outcome predictability was observed, *F*(1,42)=5.05, *p*=.030, *η^2^_p_*=.107.

Again, the analog ANOVAs was conducted to examine gaze time during the CI and the PCI separately. Notably, significant results were only observed for the CI: Main effect of outcome predictability, *F*(1,42)=5.07, *p*=.030, *η^2^_p_*=.108, as well as main effect of block, *F*(8,336)=12.00, *p*<.001, *η^2^_p_*=.222, were significant. In addition, the counterbalance factor cave condition significantly interacted with outcome predictability and block, *F*(16,336)=1.88, *p*=.021, *η^2^_p_*=.082. None of the other main effects and interactions during the CI and the PCI reached significance (largest *F*=2.44, corresponding *p*=.099).

# Experiment 4

Phase 1.

The main analysis was conducted for each group, using a 2 (outcome) × 2 (cave condition) × 12 (block) ANOVA with within-subjects factors of outcome and block. The counterbalancing factor cave condition significantly interacted with outcome and block in Group Cue-absent, *F*(14,420)=1.80, *p*=.037, *η^2^_p_*=.057.

The additional analyses using an analog ANOVA examined gaze time during the CI and the PCI for each group, respectively. They demonstrated a non-significant difference in gaze time between o1 and o2 trial during the CI for Group Outcome-absent (main effect of outcome: *F*(1,30)=4.03, *p*=.054, outcome × block interaction: *F*(23,690)=1.62, *p*=.096). In contrast, Group Cue-absent demonstrated a difference in gaze time between the two trial types during the PCI (main effect of outcome: *F*(1,30)=16.69, *p*<.001, *η^2^_p_*=.358, outcome × block interaction: *F*(14,420)=2.37, *p*=.020, *η^2^_p_*=.073). In addition, the tests revealed a significant main effect of block for Group Outcome-absent during the CI, *F*(23,690)=9.43, *p*<.001, *η^2^_p_*=.239, and a significant cave condition × outcome interaction in both groups during the CI as well as the PCI (Group Outcome-absent during the CI, *F*(1,30)=4.41, *p*=.044, *η^2^_p_*=.128, and the PCI, *F*(1,30)=15.48, *p*<.001, *η^2^_p_*=340; Group Cue-absent during the CI, *F*(1,30)=10.04, *p*=.004, *η^2^_p_*=.251, and the PCI, *F*(1,30)=9.07, *p*=.005, *η^2^_p_*=.232). No further main effects or interactions during the CI and the PCI reached significance (largest *F*=1.13, corresponding *p*=.153).

Phase 2.

Again, additional analyses examined gaze time during the CI and the PCI separately for each group, using the 2 (outcome predictability) × 2 (cave condition) × 12 (block) ANOVAs. The results suggested that the difference in learning about the novel relationships involving o1 and o2, shown by the main analysis of Group Outcome-absent, relied on longer gaze time towards the area of o2 than o1 during the PCI, *F*(1,30)=4.85, *p*=.035, *η^2^_p_*=.139 (in line with illustrations of Fig 6B). Further, the difference in gaze time between the two trial types during the PCI shown by Group Cue-absent illustrated in Figure 6d was not confirmed by the statistical test, *F*(1,30)=4.00, *p*=.055. In addition, we also observed a significant main effect of block for Group Outcome-absent during the CI, *F*(11,330)=7.53, *p*<.001, *η^2^_p_*=.201, and for Group Cue-absent during the both intervals (CI: *F*(11,330)=8.81, *p*<.001, *η^2^_p_*=.227, PCI: *F*(11,330)=4.81, *p*=.001, *η^2^_p_*=.138). None of other main effects and interactions during the CI and the PCI reached significance (largest *F*=3.55, corresponding *p*=.069).

# Experiment 5

Phase 1.

The additional analyses examined gaze time during the CI and PCI for each group, using the 2 (outcome) × 2 (cave condition) × 12 (block) ANOVAs with within-subjects factors of outcome and block. We found that the difference in learning about the two trial types revealed by the main analysis of Group Continuity relied on stronger responses in anticipation of o1 than o2 during the CI (main effect of outcome: *F*(1,30)=6.72, *p*=.015, *η^2^_p_*=.183, outcome× block interaction: *F*(23,690)=1.97, *p*=.030, *η^2^_p_*=.061). Moreover, a significant main effect of block was observed in Group Continuity during both intervals (CI: *F*(23,690)=10.34, *p*<.001, *η^2^_p_*=.256; PCI: *F*(23,667)=2.06, *p*=.046, *η^2^_p_*=.066) and in Group Reversal during the CI (*F*(14,420)=10.98, *p*<.001, *η^2^_p_*=.268). Additionally, a significant cave condition × outcome interaction was shown in both groups (Continuity during the CI, *F*(1,30)=9.42, *p*=.005, *η^2^_p_*=.239, and during the PCI, *F*(1,30)=5.07, *p*=.032, *η^2^_p_*=149; Reversal during the PCI, *F*(1,30)=6.99, *p*=.013, *η^2^_p_*=.189). No further main effects or interactions during the CI and the PCI reached significance (largest *F*=3.02, corresponding *p* =.093).

Phase 2.

The main analysis, using a 2 (outcome predictability) × 2 (Group Continuity vs. Reversal) × 2 (cave condition) × 12 (block) ANOVA with within-subjects factors of outcome predictability and block, revealed a significant cave condition × group interaction, *F*(1,60) =8.35, *p*=.005, *η^2^_p_*=.122.

The additional analyses using the analog ANOVAs examined gaze time during the CI and the PCI separately. Notably, a difference in gaze time between groups based on the prior predictability of outcomes was shown during the PCI (group × outcome predictability interaction: *F*(1,60)=4.25, *p*=.044, *η^2^_p_*=.066). Further, a significant main effect of block was observed during both intervals (CI: *F*(11,660) =16.12, *p*<.001, *η^2^_p_*=.212, PCI: *F*(11,660) =9.82, *p*<.001, *η^2^_p_*=.141). During the CI, we found a significant group × block interaction, *F*(11,660)=2.81, *p*=.004, *η^2^_p_*=.045, and a significant cave condition × group interaction, *F*(1,60)=4.46, *p*=.039, *η^2^_p_*=.069. No further main effects or interactions during the CI and the PCI were significant (largest *F*=3.69, corresponding *p*=.059).

Because of the significant group × outcome predictability interaction during the PCI, gaze time in each group during the PCI were further analyzed separately. As shown by Figs 7B and 7D, both groups reached maximum anticipation of the outcomes based on their respective cues within the first two blocks of Phase 2. Thus, these additional analyses included gaze time across Block 1 and 2. As results, we found that neither Group Continuity nor Reversal demonstrated a difference in gaze time between two trial types (main effect of outcome predictability for Continuity, *F*(1,30)=2.92, *p*=.098, *η^2^_p_*=.089, and for Reversal, *F*(1,30) =3.43, *p*=.074, *η^2^_p_*=.102). The analyses revealed a significant main effect of block in Group Continuity, *F*(1,30) =9.33, *p*=.005, *η^2^_p_*=.237, and a significant main effect of cave condition in Group Reversal, *F*(1,30) =5.76, *p*=.023, *η^2^_p_*=.161. None of other main effects or interactions during the CI and the PCI reached significance (largest *F*=3.63, corresponding *p*=.067).

# Experiment 6

Phase 1.

The main analysis, using an 2 (outcome) × 2 (cave condition) × 14 (block) ANOVA with within-subjects factors of outcome and block, revealed a significant cave condition × block interaction, *F*(13,377)=2.77, *p*=.008, *η^2^_p_*=.087.

The additional analyses using the analog ANOVAs examined gaze time during the CI and the PCI separately. We found that the difference in dwell time between the two trial types revealed by the main analysis was based on a difference in responses during the PCI (main effect of outcome: *F*(1,29)=17.65, *p*<.001, *η^2^_p_*=.378; outcome × block interaction: *F*(13,377)=2.62, *p*=.015, *η^2^_p_*=.083). In addition, a significant main effect of block was shown during the CI, *F*(13,377)=9.97, *p*<.001, *η^2^_p_*=.256. No other main effects or interactions during the CI and the PCI were significant (largest *F*=3.56, corresponding *p*=.069).

Phase 2.

The main analysis, using a 2 (outcome predictability) × 2 (cave condition) × 10 (block) ANOVA with within-subjects factors of outcome predictability and block, showed a significant cave condition × outcome predictability × block interaction, *F*(9,270)=2.61, *p*=.019, *η^2^_p_*=.080.

The additional analyses using the analog ANOVAs examined gaze time during the CI and the PCI separately. They did not reveal any difference in gaze time based upon the prior predictability of outcomes. In addition, we observed a significant cave condition × outcome predictability interaction during both intervals (CI: *F*(1,30) =7.48, *p*=.010, *η^2^_p_*=.200, PCI: *F*(1,30)=7.69, *p*=.009, *η^2^_p_*=.204), a significant main effect of block during the CI, *F*(9,270) =4.23, *p*=.002, *η^2^_p_*=.124, as well as a significant cave condition × outcome predictability × block interaction during the CI, *F*(9,270) =2.37, *p*=.014, *η^2^_p_*=.073. None of the other main effects and interactions during the CI and the PCI were significant (largest *F*=2.02, corresponding *p*=.083).
